# microRNA-146a inhibits cancer metastasis by downregulating VEGF through dual pathways in hepatocellular carcinoma

**DOI:** 10.1186/1476-4598-14-5

**Published:** 2015-01-21

**Authors:** Zheng Zhang, Yang Zhang, Xiu-Xuan Sun, Xi Ma, Zhi-Nan Chen

**Affiliations:** Cell Engineering Research Center & Department of Cell Biology, State Key Laboratory of Cancer Biology, Fourth Military Medical University, Xi’an, 710032 P. R. China

**Keywords:** Hepatocellular carcinoma, MicroRNA-146a, VEGF, HAb18G/CD147, Invasion, Metastasis

## Abstract

**Abstract:**

Growing evidence indicates that miR-146a is involved in carcinogenesis and tumor progression in several human malignancies. However, the molecular details underlying miR-146a mediated regulation of its target genes and its precise biological function in cancer, especially in hepatocellular carcinoma (HCC) remains unclear.

**Methods:**

The expression levels of genes including miR-146a, APC, VEGF and HAb18G were examined in HCC cell lines and patient specimens were compared with control levels using quantitative reverse transcription-PCR. The functions of miR-146a and HAb18G in migration/invasion and liver metastasis formation were determined by transwell and spleen injection assays, respectively. miR-146a related genes were determined by PCR array. The potential regulatory targets of miR-146a were determined by bioinformatics and prediction tools, correlation with target protein expression, and luciferase reporter assay. DNA methylation status of miR-146a promoter were performed by PCR analysis of bisulfite-modified genomic DNA.

**Results:**

We demonstrated that miR-146a expression was markedly downregulated in hepatoma cells and hepatoma tissues compared to immortalized normal liver epithelial cells and normal hepatic tissues. DNA methylation of miR-146a promoter correlated with its downexpression and with liver cancer metastasis. The restoration of miR-146a dramatically suppressed HCC cell invasion and metastasis by repressing VEGF expression through upregulating APC, which inhibits β-catenin accumulation in nucleus, and downregulating NF-κB p65 by targeting HAb18G. In human HCC, miR-146a expression was negative correlated with increased HAb18G, VEGF, NF-κB p65 and beneficial prognosis.

**Conclusion:**

This study identified a novel target of miR-146a and defined miR-146a as a crucial tumor suppressor in human HCC that acts through multiple pathways and mechanisms to suppress HCC invasion or metastasis.

**Electronic supplementary material:**

The online version of this article (doi:10.1186/1476-4598-14-5) contains supplementary material, which is available to authorized users.

## Introduction

Hepatocellular carcinoma (HCC) is one of the most common malignancies and one of the leading causes of cancer-related death worldwide [1, 2]. Despite recent advances in disease management and treatment, HCC patients still have a very dismal long-term prognosis. For advanced-stage HCC patients, the overall 5-year survival rate is less than 5% [3]. The main challenges in the treatment of HCC involve intrahepatic recurrence and metastasis, which simultaneously predict poor outcome for HCC patients. The identification of critical players that suppress these processes may lead to novel therapeutic targets for improving the prognosis of these patients. Various previous studies have identified some key signaling transduction cascades that are implicated in the progression, invasion, and metastasis of HCC, such as the EGFR/PI3K pathway [4], the RhoGTPase/Rho-effector pathway [5], the SAPK/JNK pathway [6], and the Ras/MAPK pathways [7]. However, the underlying molecular mechanisms of HCC metastasis are far from fully understood.

In the past decade, increasing attention has been paid to the important biological and pathological roles of microRNAs (miRNAs), a type of small conserved RNA molecule of approximately 17–22 nucleotides in length [8, 9]. As post-transcriptional regulators of gene expression, these small non-coding RNAs complementarily bind to the 3′ untranslated region (3′-UTR) of their target messenger RNAs (mRNAs), leading to either the degradation of mRNAs or the inhibition of protein translation. miRNAs are involved in the regulation of most cellular processes, including cell proliferation, migration, and apoptosis [10]. In recent years, numerous studies have shown that aberrant expression of miRNAs is associated with the development and progression of various types of cancer, including HCC [10], and some of these miRNAs function as tumor suppressor genes or oncogenes [11, 12]. MiR-146a is located on human chromosome 5q34, which is a region that is often deleted in human tumors [13], and has been reported to be aberrantly expressed in several cancers. For example, it is downregulated in breast [14], hormone-refractory prostate cancer [15], and pancreatic cancer [16], which indicates that miR-146a is a potential tumor suppressor. MiR-146a has also been shown to inhibit cancer cell metastasis by targeting IRAK-1 [17–19]. However, it remains unknown whether miR-146a is involved in the regulation of HCC cell invasion and how this miRNA mediates invasive inhibition.

In this study, we discovered that miR-146a is downregulated in human HCC and likely suppresses hepatoma cell invasion and metastasis by downregulating VEGF expression through 2 signaling pathways. First, miR-146a mediates the upregulation of APC, which leads to the inhibition of the nuclear accumulation of β-catenin, and second, this miRNA inhibits HAb18G/CD147 (HAb18G) expression, a direct target of miR-146a, which consequently downregulates NF-κB p65. These findings provide a framework for better understanding the pathogenesis of HCC.

## Materials and methods

### Ethics statement and clinical specimens

Frozen and paraffin-embedded tissues from 53 HCC cases were collected from XiJing Hospital of Fourth Military Medical University, with patients provided prior consent and approval of Fourth Military Medical University. The study protocol was approved by the Ethics Committee of the Fourth Military Medical University. The sample enrollment criteria included those with HCC diagnosed by two independent pathologists, detailed information on clinical presentation and detailed follow-up data for at least 3 years. All patients had been treated with a combination of surgery and platinum-based chemotherapy.

### DNA isolation and bisulfite treatment and BS-PCR DNA Sequencing

DNA was isolated using the QIAGEN DNAesasy Blood and Tissue kit following the guidelines of the manufacturer. DNA was modified sodium-bisulfite using the EpiTect Bisulfite Kit (QIAGEN) according to the manufacturer’s instructions. The CpG sites of miR-146a promoter region were amplified by PCR containing bisulfite DNA template, Hotstartaq, bisulfite specific primers following established procedure. The detail information of primers is listed in Additional file 1: Table S1. After PCR reaction. DNA fragment were purified using the Cycle pure kit from Omega bio-tek. The purified PCR fragments were cloned into pGEM-T easy vector (Promega), and individual clones were sequenced.

### Cell culture and nuclear-cytoplasmic fractionation

The human HCC cell lines SMMC-7721, Huh-7, and HepG2 and the human embryonic kidney cell line 293 T (HEK 293 T) were purchased from Shanghai Institute for Biological Sciences (Shanghai, China) and routinely cultured in Dulbecco’s Modified Eagle Medium (DMEM) (GIBCO BRL, Grand Island, NY) containing 10% fetal bovine serum (Hyclone Laboratories, Logan, UT) at 37°C in a humidified atmosphere of 5% CO_2_. Nuclear-cytoplasmic fractionation was performed using Nuclear and Cytoplasmic Extraction Reagents (Beyotime, Wuhan, China) according to the manufacturer’s protocol.

### RNA extraction and quantitative real-time polymerase chain reaction (qPCR)

Total RNA was isolated from cultured cell lines and tissue samples using TRIzol Reagent (Invitrogen, CA), according to the manufacturer’s instructions. For the enrichment of normal liver cells, paraffin-embedded normal liver tissue samples from 11 hepatic hemangioma patients were microdissected on HE-stained sections using laser capture microdissection under a microscope (Leica, LMD7000), according to the method described previously [20]. Normal liver tissues were also collected, and subsequent RNA isolation was performed using the NucleoSpin® FFPE RNA kit (Macherey-Nagel) according to the manufacturer’s protocol.

Reverse transcription (RT) reactions were performed using the Prime-Script RT reagent kit (TaKaRa, Dalian, China). The qPCR was performed using a SYBR Premix Ex Taq™ II kit (Takara). For miR-146a detection, mature miR-146a was reverse transcribed, with specific RT primers that were designed as previously described [21], and normalized by RUN6. The sequences of the PCR and RT primers are listed in Additional file 1: Table S1.

### Pathway-specific expression array

The human tumor metastasis RT^2^ profiler PCR array PAHS-028Z (QIAGEN) was used to assess the effect of miR-146a on the expression of 84 known metastasis-related genes. Total RNA was isolated either from miR-146a-transfected SMMC-7721 cells or from negative control-transfected SMMC-7721 cells using the HP Total RNA kit (Omega bio-tek). Total RNA was reverse transcribed into cDNA using the RT2 First Strand Kit (QIAGEN) with RT2 qPCR master mix containing SYBR Green (QIAGEN), according to the supplier’s instructions. The real-time PCR reaction was performed with a CFX384 real-time system (BIO RAD). Four reference genes with the lowest standard deviations across replicates were used in the analysis (B2M, GAPDH, HPRT1, and RPLP0). Expression profiles were obtained from 3 independent experiments.

### Cell apoptosis and cell cycle analysis

For cell apoptosis analysis, the cells were resuspended in 1× Binding Buffer, and 5 μl of Annexin FITC Conjugate and 10 μl of Propidium Iodide Solution were separately added to each cell suspension. The stained cells (1 × 10^5^) were then analyzed using a FACScalibur flow cytometer (BD Biosciences).

For cell cycle analysis, the cells were harvested by trypsinization, washed in ice-cold phosphate-buffered (PBS), and then fixed in 70% ice-cold ethanol. The cells were washed with PBS and resuspended in Staining Solution (50 μg/mL propidium iodide, 1 mg/mL of RNase A, and 0.1% Triton X-100 in PBS). Cell cycle profiles of 2 × 10^5^ cells were analyzed using a FACScalibur flow cytometer (BD Biosciences).

### Cell proliferation assay

SMMC-7721, HepG2, and Huh-7 cells were plated in 96-well plates at 3 × 10^3^ cells per well after transfection with miR-146a or anti-miR-146a for 48 h. Cells were cultured for 24, 48, 72, and 96 h, and then the absorbance at 450 and 630 nm was measured after incubation with 10 μl of WST-1 (Roche Applied Science, Indianapolis, IN, USA). Each assay was performed in triplicate.

### Immunofluorescence analysis

Cells were fixed in 4% paraformaldehyde at room temperature for 15 min, followed by permeabilization in 1 × PBS containing 0.2% Triton X-100 for 10 min at room temperature. The cells were blocked in blocking solution (1 × PBS containing 10% normal goat serum) for at least 2 h. After being briefly washed, the cells were incubated with a rabbit anti human β-catenin or NF-κB p65 antibody at 4°C for overnight. After being washed, a goat anti-rabbit IgG conjugated with Alexa Fluor 488 (Invitrogen) was incubated with the cells at room temperature for 1 h. DAPI (Sigma) was used to stain the DNA. The fluorescence images were acquired using a laser scanning microscope (A1, Nikon, Japan).

### Immunohistochemical staining

Fresh-frozen tissue samples were formalin-fixed, paraffin-embedded, and then cut into 5-μm sections. Immunohistochemical staining for the HAb18G [22], APC and VEGF was performed, and the expression level was independently evaluated by 2 senior pathologists according to the proportion and intensity of positive cells. A score of 0 (no staining), 1 (any percentage with weak intensity or <30% with intermediate intensity), 2 (>30% with intermediate intensity or <50% with strong intensity) or 3 (>50% with strong intensity) was assigned to each sample.

### Vector construction

The expression vectors for miR-146a (pcDNA3.1-miR-146a) and HAb18G (pcDNA3.1-HAb18G) were constructed by cloning the miR-146a precursor sequences amplified from the genomic DNA of SMMC 7721 cells and the HAb18G open reading frame (ORF) sequence (without 3′-UTR) amplified from the total RNA of SMMC 7721 cells into the multiple cloning site of the mammalian expression vector pcDNA3.1 (Invitrogen, CA). In addition, we generated 2 luciferase reporter vectors with a wide-type (pGL3-HAb18G wt) or mutant (pGL3-HAb18G mut) 3′-UTR fragment of HAb18G inserted downstream of the stop codon of the firefly luciferase gene in the pGL3 vector (Promega). The 3′-UTR fragment of HAb18G for both pGL3-HAb18G wt and pGL3-HAb18G mut contained putative binding sites for miR-146a, whereas the mutant 3′-UTR of pGL3-HAb18G mut carried a mutated sequence in the complementary site for the seed region of miR-146a. The sequences of all primers used in vector construction are provided in Additional file 1: Table S1. All constructed vectors were confirmed by direct sequencing.

### Wound migration and invasion assay

Cells transfected with miR-Ctrl/miR-146a, or nonrelative control moleculars (NC)/antagomiR-146a were plated in 24-well culture plates at a density of 10^5^ or 2 × 10^5^ (Huh-7, hepG2) cells per well, and incubated for 24 h or 48 h (Huh-7, hepG2) to reach confluence. Using a 200 μl tip, a wound was made in the monolayer (at time 0). The cells were then washed with PBS and incubated. The distance between the two sides of the wound was measured with an Olympus CX71 microscope (Olympus). The distance between the two sides of the wound after 20-72 h of migration was divided from the distance at time 0 and represented on a graph.

The invasion abilities of cultured cells were measured using an *in vitro* transwell assay with modified Boyden chambers containing polycarbonate filters (Millipore, MA), according to the manufacturer’s instructions. Cells transfected with miR-146a/miR-Ctrl, or antagomiR-146a/nonrelated control molecules (NC) were plated 24 h after transfection in serum-free medium and allowed to invade towards a 10% FBS medium for 24 h, or 48 h. Cells that remained on top of the filter were scrubbed off, and those that invaded the underside of the filter were fixed and stained with crystal violet.

### Generation of SMMC-7721-miR-146a stable cell lines

Pre-miR-146a were amplified by PCR using cDNA from SMMC-7721 cells and cloned into pcDNA3.1 vector. The pcDNA-miR-146a and the empty vector alone were transfected into SMMC-7721 cells using lipofectamine 2000 (Invitrogen). At 48 h post-transfection the cells were culture in complete medium with 400 μg/ml G418 for 4 weeks.

### In vivo metastasis assay

An experimental metastasis model in athymic nude mice was developed using the HCC cell line SMMC-7721, which has relatively strong *in vivo* invasive and metastatic properties, as previously described [23]. Briefly, *nude* mice were anesthetized with pentobarbital and a transverse incision was made in the left flank through the skin and peritoneum. The spleen was carefully exposed and 2 × 10^6^ viable SMMC-7721 cells transfected with pcDNA3.1 or pcDNA-pre-miR-146a were injected under the spleen capsule via a 27-gauge needle. Six weeks after the injection, the mice were sacrificed under anesthesia and tumor metastasis was examined under a stereo microscope.

### Luciferase reporter assay

The 3′-UTRs of *Ehmt2*, *mpzl1*, *rhoA*, *kif2c*, and *HAb18G* were amplified by PCR and cloned downstream of the *luciferase* gene in the pGL3 reporter vector (Promega). Cells (3 × 10^4^) were seeded in triplicate in 24-well plates and allowed to settle for 24 h. Then, approximately 100 ng of pGL3-HAb18-3′-UTR (wt or mut) and 1 ng of pRL-TK Renilla plasmid (Promega) were transfected into cells using Lipofectamine 2000 (Invitrogen) according to the manufacturer’s recommendations. Luciferase and Renilla signals were measured 48 h after transfection using the Dual Luciferase Reporter Assay Kit (Promega) according to the manufacturer’s protocol. Three independent experiments were performed, and the data are presented as the mean ± SD.

### Western blotting

Western blotting analysis was performed according to the standard protocol described previously [22].

The samples were subjected to SDS-PAGE and transferred onto a polyvinylidene fluoride membrane. The primary antibodies used in this study were as follows: anti-HAb18G (1:4,000 [24], prepared by our lab), rabbit-anti-β-catenin (1:500, Santa Cruz), rabbit-anti-APC (1:500, Boster), rabbit-anti-VEGF (1:400, Boster), rabbit-anti-NF-κB p65 (1:400, Boster), and an anti-α-tubulin antibody as a loading control. The secondary antibodies used were either goat anti-mouse or goat anti-rabbit IgG (PIERCE), depending on the primary antibody used.

### Statistical analysis

Statistical significance was evaluated using the Student’s t-test for paired comparisons. All values are expressed as the mean ± SD. *P* values <0.05 (using a 2-tailed paired t-test) were considered to indicate significantly significant differences between 2 groups of data. Non-metastasis time data were represented using Kaplan Meier curves and differences were compared by means of the pairwise log-rank test.

## Results

### MiR-146a is frequently down-regulated in HCC and associated with tumor invasion and metastasis

**Figure 1:**
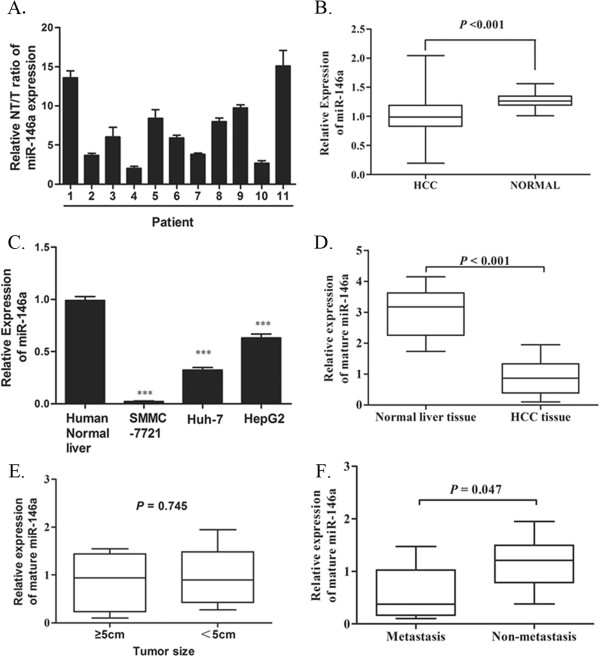
**Downregulation of miR-146a in human HCC cell lines and tissues. A**. miR-146a expression was examined in paired primary HCC tissues (T) and their match adjacent non-tumor tissues (NT) of 11 individual patients. The miR-146a levels were normalized to *RUN6* expression. **B**. miR-146a is decreased in HCC tissues compared with in matched non-tumor liver tissues (n = 96; NCBI/GEO/GSE 22058). P value was calculated by Student’s test. **C**. and **D**. Real-time RT-PCR analysis of miR-146a expression in human normal liver tissues and HCC cell lines **(C)**., including SMMC-7721, Huh-7 and HepG2, and in 53 HCC cases **(D)**. miR-146a levels were normalized to *RUN6* expression. P value was calculated by Student’s test. **E**. Real-time RT-PCR analysis of miR-146a expression in 28 big HCC tissue samples (≥ 5 cm) and in 15 small HCC tissue samples (< 5 cm) was determined by real-time RT-PCR and normalized to the level of RUN6. P value was calculated by Student’s test. **F**. Real-time RT-PCR analysis of miR-146a expression in 26 HCC tissue samples with metastasis and in 17 HCC tissue samples without metastasis was determined by real-time RT-PCR and normalized to the level of RUN6. P value was calculated by Student’s test.

In attempt to explore that miR-146a expression levels differ between tumor and non-tumor tissues, we examined the expression in 11 pairs of HCC tissues and matched tumor adjacent tissues. As shown in Figure 1A, the comparative analysis indicated that miR-146a was differentially downregulated in all 11 examined tumor tissues paired with adjacent non-tumor tissues from the same patient. Consistent with our results, published microarray data have also shown that miR-146a is downregulated in HCC tissues as compared to matched non-cancer liver tissue (Figure 1B, n = 96, NCBI/GEO/GSE22058). Then, we detected miR-146a expression in a panel of cell lines (SMMC-7721, Huh-7, and HepG2) and in a cohort of HCC patients (n = 53) and normal liver samples (n = 11), and observed that miR-146a was downregulated in all 3 HCC cell lines as compared to normal liver cells that were microdissected from 7 normal liver samples (Figure 1C), similarly, miR-146a level was downexpressed in HCC samples compared to those in normal liver samples (Figure 1D). Altogether, these results indicate that miR-146a is downregulated in HCC. The human HCC cell line SMMC-7721, which has negligible levels of endogenous miR-146a, was found suitable for miR-146a overexpression studies. Because miR-146a expression levels in the Huh-7 and HepG2 cell lines were much higher than in SMMC-7721 cells, the miR-146a inhibition studies were performed in these cell lines. Furthermore, the relationship between the expression of miR-146a and the clinical characteristics of HCC patients was analyzed, which showed that miR-146a expression is unrelated with tumor size, however, the level of miR-146a is remarkably lower in HCC patients with metastasis (n = 26) than in those without (n = 17) (Figure 1E and F). All together, these results suggesting the important roles of miR146a in pathogenesis of HCC and prognosis of HCC patients.

### Promoter methylation mediates decrease of miR-146a expression

**Figure 2:**
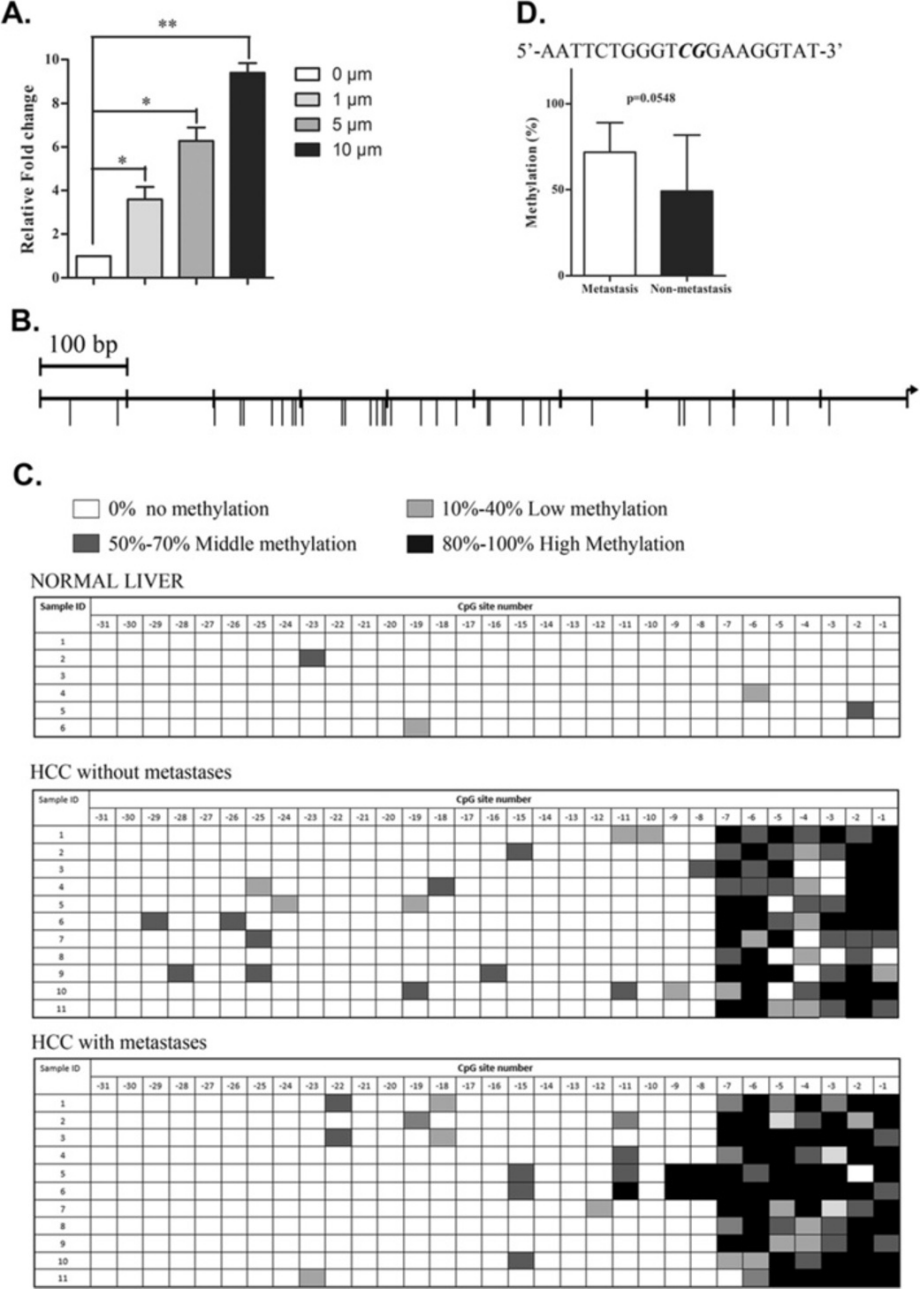
**The association between the expression and promoter methylation of miR-146a in human liver cancer tissues. A**. Real-time RT-PCR analysis of miR-146a expression in SMMC-7721 cells treated with 5-aZa-dC for 24 h. Error bars represent ± SD. A student t-test was employed. (*, p < 0.05) (**, p < 0.01). **B**. Diagram of the locations of the CpG sites. Vertical bars represent CpG sites in the promoter of miR-146a.**C**. Bisulfite genomic sequencing analyses of the miR-146a CpG site in normal liver tissues (n = 6) and HCC tissues (n = 22). The filled and open boxes are represented methylated and unmethylated CpG, respe60ctively. Ten single clones are represented for each sample. Open boxes, 0% methylation; light filled boxes, 0-50% methylation; black filled boxes, 50-100% methylation. NL, normal liver; HM, HCC with metastasis; HN, HCC without metastasis. **D**. the association between methylation status of candidate position and HCC metastasis. Error bars represent ± SD.

As miR-146a was found to be the markedly down-regulation in HCC. Firstly, we ruled out the possibility that the gene copy number alternations in HCC genome using real-time PCR (data not shown). Then, we performed a demethylation experiment by treating HCC cancer cells with 5-azaC and found an induction of miR-146a (Figure 2A), which indicating DNA methylation is likely to regulate miR146a expression in the liver. Furthermore, we analyzed the methylation status of the miR-146a promoter in HCC patient tumors (n = 22) and normal liver tissues (n = 5). We found that 7 hypermethylated CpG sites adjacent transcription start site in the promoter region of miR-146a may in HCC compared with those in normal liver tissues though bisulfate sequencing (Figure 2B and C), and one of seven site displayed trends of negative correlation between methylation and tumor metastasis (p = 0.0548 for site 4, Figure 2D) .

### MiR-146a inhibits HCC invasion and metastasis

**Figure 3:**
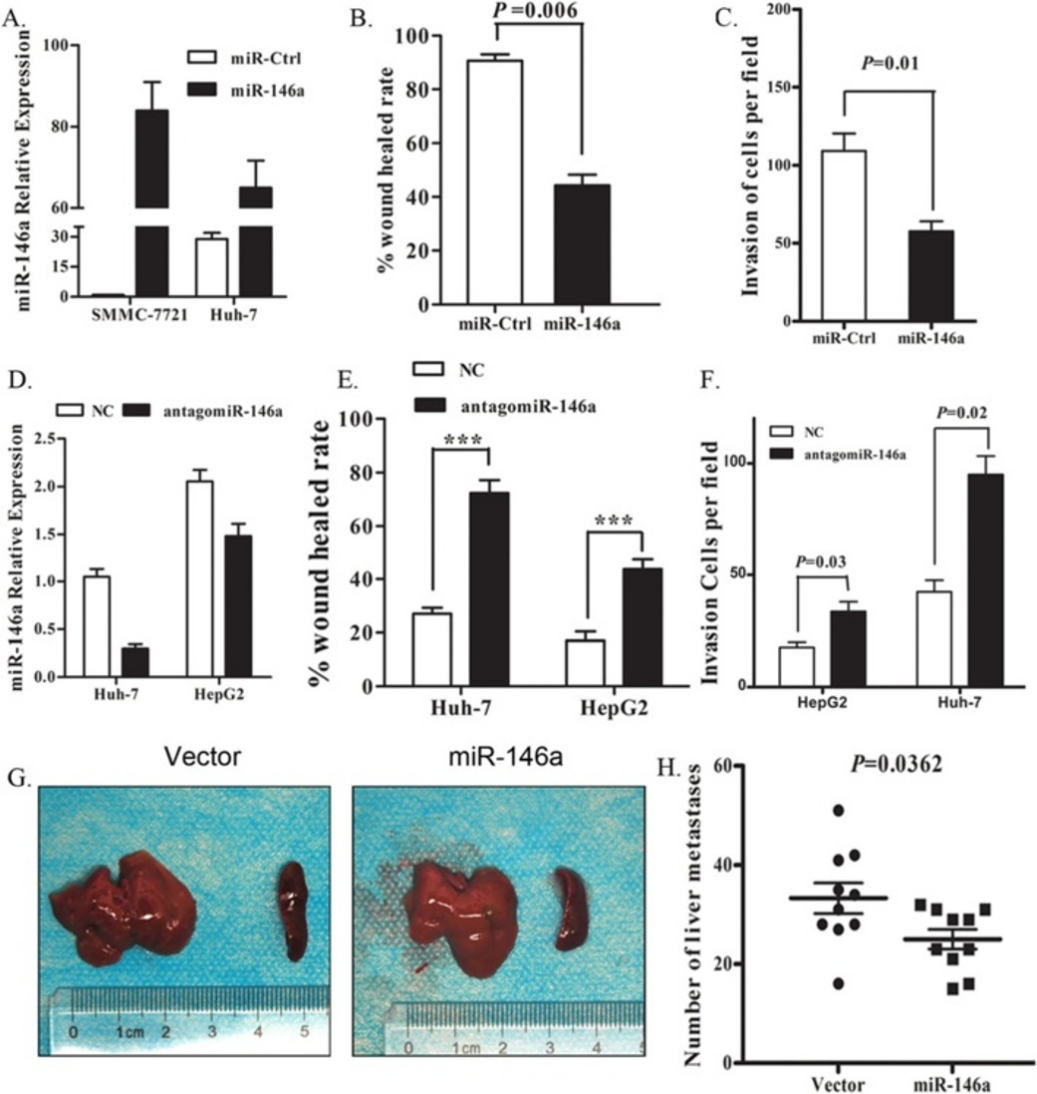
**miR-146a inhibited cell invasion and metastasis** ***in vitro*** **and** ***in vivo*** **. A**. Changes in miR-146a levels in SMMC-7721 and Huh-7 cells transfected with miR-146a or miRNA control (miR-Ctrl) for 48 h as measured by real-time RT-PCR. the datum represents the mean ± SD. **B**. Quantification of wound healing assay of SMMC-7721 cells transfected with miR-146a or miRNA control (miR-Ctrl). the datum represents the mean ± SD. *p < 0.05. **C**. *In vitro* invasion assay. Cells from the same transfection as in **(B)**. were seeded into triplicate mitrigel coated invasion chambers at 24 h post-transfection and allowed to invade toward serum for 24 h. The invading cell numbers on each filter were counted and data were plotted. the datum represents the mean ± SD. The student’s t test was used to compare the difference between two groups. *p < 0.05. **D**. Changes in miR-146a levels in Huh-7 and HepG2 cells transfected with antagomiR-146a or nonrelated control moleculars (NC) for 48 h as measured by real-time RT-PCR. the datum represents the mean ± SD. **E**. Quantification (right) of wound healing assay of Huh-7 and HepG2 cells transfected with antagomiR-146a or nonrelated control moleculars (NC). The student’s t test was used to compare the difference between two groups. the datum represents the mean ± SD. *p < 0.05. **F**. *In vitro* invasion assay. Cells from the same transfection as in (E) were seeded into triplicate mitrigel coated invasion chambers at 24 h post-transfection and allowed to invade toward serum for 48 h. The invading cell numbers on each filter were counted and data were plotted. the datum represents the mean ± SD. The student’s t test was used to compare the difference between two groups. *p < 0.05. **G**. Representative images of the liver, spleen and number of visible liver metastases in vector- or miR-146a treated mice. Nude mice were injected under the spleen capsule with 2 × 10^6^ vector or miR-146a stable transfected SMMC-7721 cells via a 27-gauge needle (n = 10 in each group). **H**. Tumor nodule number were counted (n = 10 in each group). Data are mean + SD. * p < 0.05.

Given the low level of miR-146a expression detected in HCC tissues, we asked whether miR-146a may function as a tumor suppressor and could affect cell migration or invasion. To address this question, we transfected miR-146a into SMMC-7721 and Huh-7 cells (Figure 3A). Restoration of miR-146a expression in HCC cell line reduce the migration of SMMC-7721 cells, as detected in scratch migration assays (Figure 3B and Additional file 2: Figure S1A). Furthermore, the invasion rate of cells was repressed by at least 60% in the presence of miR-146a as compared to a negative control (Figure 3C and Additional file 2: Figure S1D). Cell cycle and apoptosis analysis showed that this effect was not due to the inhibition of the cell cycle or increased apoptosis (Additional file 3: Figure S2A, S2B). WST-1 analysis and PCNA expression results also showed that the ectopic transfection of miR-146a did not affect cell proliferation (Additional file 3: Figure S2C, S2D). Similar results were found in Huh-7 cells (data not shown). Furthermore, we took advantage of the fact that miR-146a is expressed in Huh-7 and HepG2 cells and performed antagomiR-mediated loss of functional experiments (Figure 3D), and found that the migration of these cells increased by 39% and 18% (Figure 3E, Additional file 2: Figure S1B, and S1C), and the invasiveness of these cells increased by 41% and 11%, respectively (Figure 3F, Additional file 2: Figure S1E). These results demonstrate that miR-146a may function as a tumor suppressor *in vitro*.

In order to test the antitumor effect of miR-146a *in vivo*, we prepared a cell line SMMC-7721-146a (7721-146a), which stable expression of miR-146a (Additional file 2: Figure S1F). Then, we injected 7721-146a or wild-type SMMC-7721 cells transfected with the blank vector (named as vector) into the spleen of nude mice. Four weeks later, a 58.69% decrease in the number of macroscopically visible liver metastases was achieved in 7721-146a group. An average of 33.3 ± 3 visible metastases nodules were counted in mice implanted with vector group, and an average of 25 ± 1.9 visible metastases per mouse was observed in mice bearing 7721-146a cells (*P* <0.001, Figure 3H). Examination of the lungs revealed an average of 10 ± 1 visible lesions in mice injected with vector group, whereas mice treated with 771-146a exhibited an average of 6 ± 1 macroscopic lung metastases (Additional file 2: Figure S1G). Altogether, these results indicate that miR-146a can inhibit HCC cell migration and invasion both *in vitro* and *in vivo*.

### Overexpression of miR-146a upregulates APC and downregulates VEGF in HCC cells

**Figure 4:**
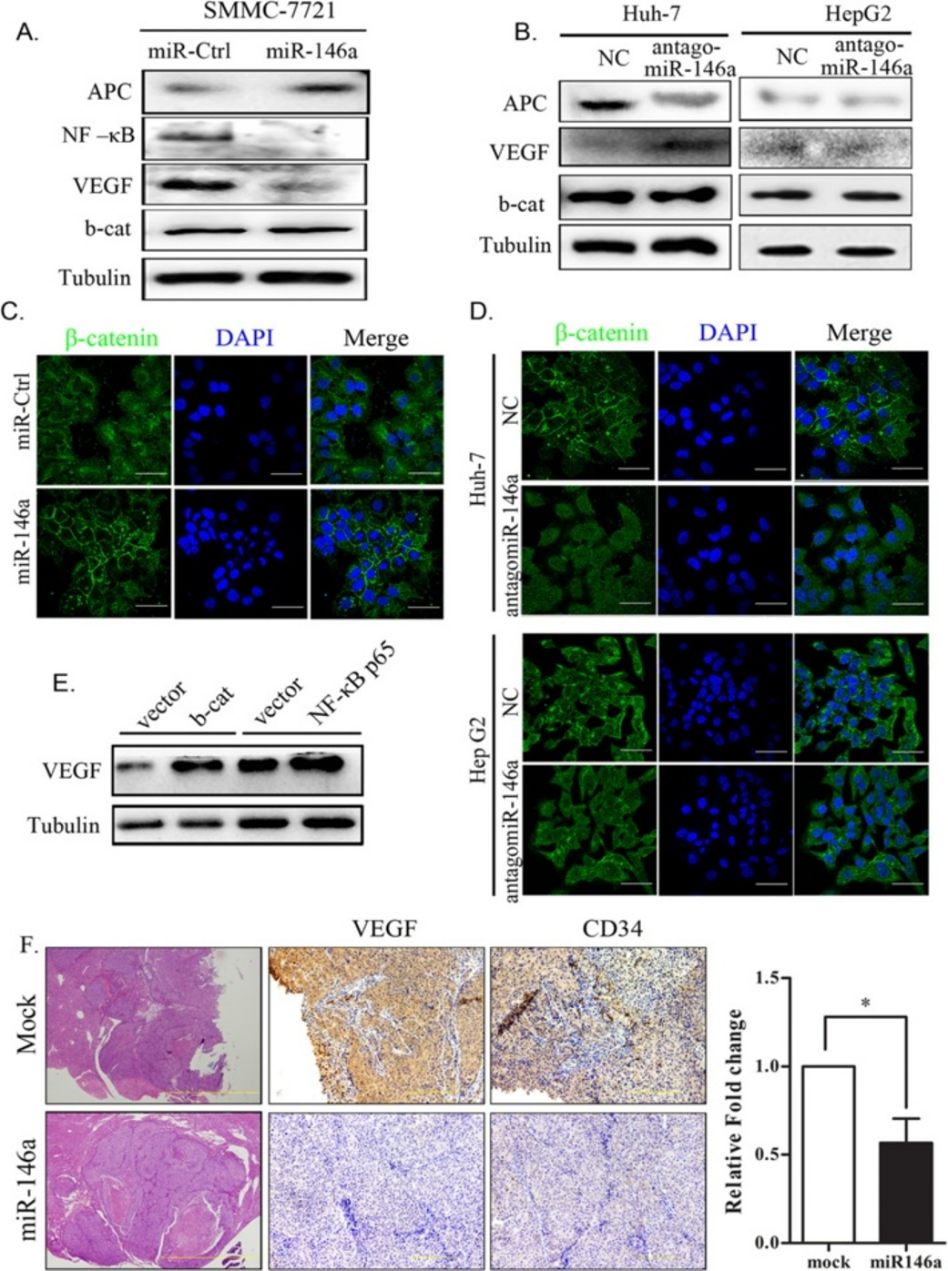
**Overexpression of miR-146a enhance APC expression and inhibit VEGF expression. A**. Western blotting analysis of APC, NF-κB p65, VEGF and β-catenin (b-cat) in SMMC-7721 cells transfected with miR-146a or miRNA control (miR-Ctrl). **B**. Western blotting analysis of APC, VEGF and β-catenin (b-cat) in Huh-7 and HepG2 cells transfected with antagomiR-146a or negative control (NC). **C**. β-catenin staining of SMMC-7721 cells transfected with miR-146a or miRNA control (miR-ctrl) for 48 h. Cell nuclei were stained with DAPI. Scale bars represent 50 μm. **D**. β-catenin staining of Huh-7 and HepG2 cells transfected with antagomiR-146a or negative control (NC) for 48 h. Cell nuclei were stained with DAPI. Scale bars represent 50 μm. **E**. Western blotting analysis of β-catenin and NF-κB p65 expression in SMMC-7721 cells transfected with pcDNA3-β-catenin or pcDNA3- NF-κB p65, respectively. **F**. Representative images of H&E staining, and VEGF and CD34 staining by immunostaining in SMMC-7721 xenograft tumors; quantitation of the number of microvessels in 10 representative hpf’s (*right*); the datum represents the mean ± SD from 6 independently treated tumors (P = 0.0258). Scale bar represents 100 μm.

To understand the potential of miR-146a to inhibit metastasis, we analyzed the expression patterns of 84 metastasis-associated genes by RT^2^ profiler PCR array (QIAGEN) in SMMC-7721 cells transfected with miRNA control (miR-Ctrl) or miR-146a. MiR-146a overexpression significantly increased *adenomatous polyposis coli (APC)* levels, while *VEGF* was downregulated by miR-146a (Additional file 4: Figure S3 and Figure 4A). To further confirm these findings and to determine whether the observed response was specific to miR-146a, we determined the expression levels of *APC* and *VEGF* in HepG2 and Huh-7 cells transfected with antagomiR-146a. We observed the downregulation of *APC* and the induction of *VEGF* at 48 h after antagomiR-146a treatment in Huh-7 cells, however, only a slight changes were detected in HepG2 cells (Figure 4B). We think about this results is due to cell specific characters.

**Figure 5:**
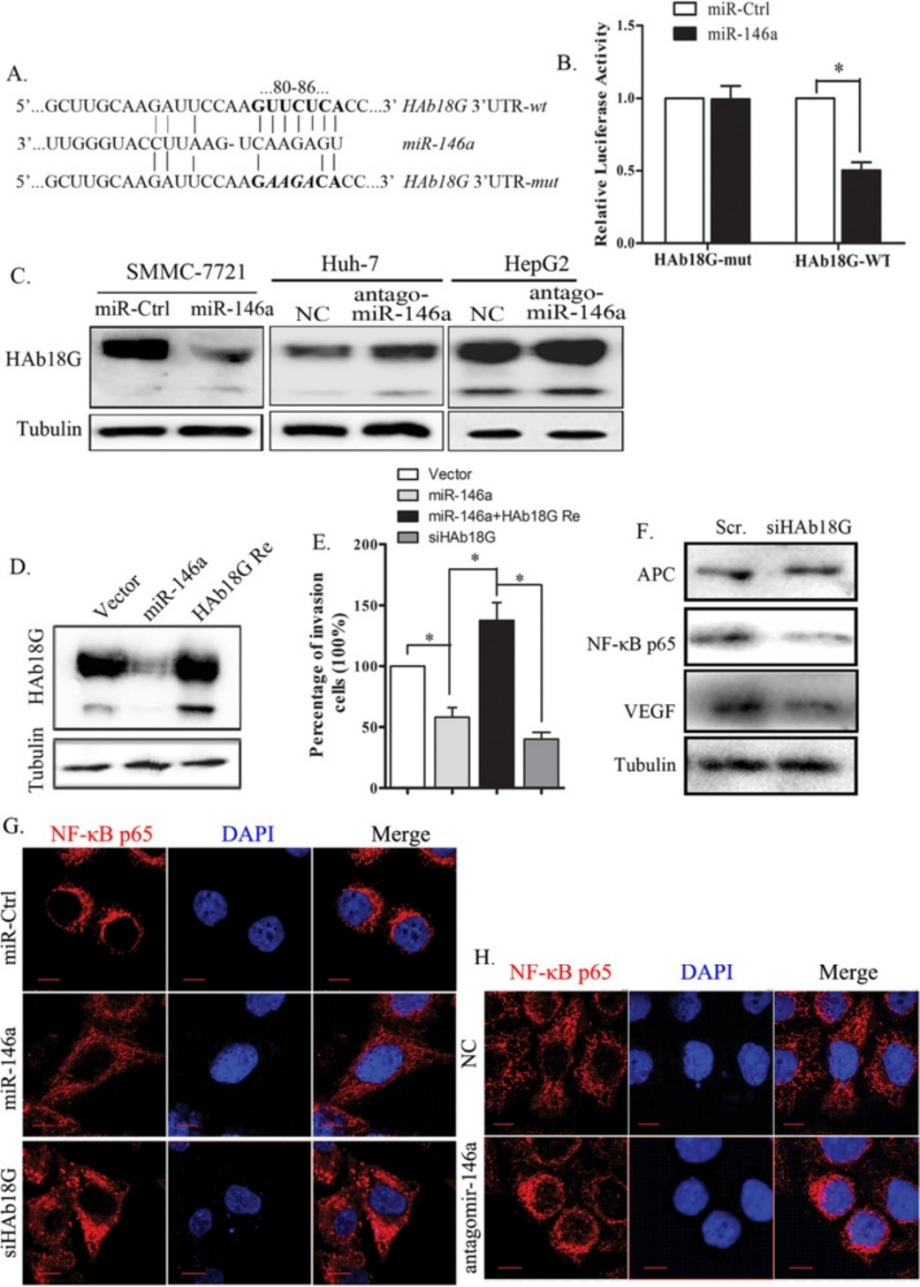
**HAb18G is a potential target of miR-146a. A**. Multiple species sequence alignment of the HAb18G/CD147 3′UTR including the putative miR-146a target site sequence (upper). The pGL3- HAb18G reporter gene has the full length of HAb18G 3’-UTR cloned into pGL3 vector. The pGL3- HAb18G -MUT vector has the four miR-146a binding sites mutated and confirmed by sequencing. SMMC-7721 were transfected with pGL3- HAb18G or pGL3-HAb18G -MUT, respectively, together with miR-146a mimics or mimic negative control. The four predicted binding sites of miR-146a were shown in the HAb18G 3′-UTR region. **B**. The relative luciferase activities were shown from three independent experiments. Error bars represent ± SD. **C**. Western blotting analysis of CD147 in SMMC-7721 cells transfected with miR-146a or control miRNA (miR-Ctrl) (left panel). Huh-7 and HepG2 cells were transfected with antago miR-146a or negative control (NC) (middle and right panel). **D**. Western blotting analysis of CD147 in SMMC-7721-miR-146a cells transfected with CD147 without 3′-UTR region (CD147 Re). **E**. *In vitro* invasion assay of SMMC-7721 cells transfected with CD147 siRNA for 24 h. The invading cell numbers on each filter were counted and data were plotted in fold change by defining the number from control cells a 100%. Error bars represent ± SD. **F**. Western blotting analysis of APC, NF-κB p65 and VEGF in SMMC-7721 cells transfected with HAb18G siRNA or scramble control. **G-H**. NF-κB p65 staining of SMMC-7721 cells transfected with miR-146a, CD147 siRNA or miR-Ctrl (G), or Huh-7 cells transfected with antagomiR-146a and NC (H). Cell nuclei were stained with DAPI. Scale bars represent 50 μm.

Because APC, a tumor suppressor, has been reported as a negative regulators of β-catenin localization [25, 26], and β-catenin can promote *VEGF* transcription [27]. In addition, NF-κB also promotes VEGF expression [28, 29]. Therefore, we hypothesized that miR-146a could inhibit VEGFA expression through a dual signaling pathway model via β-catenin and NF -κB. To test this hypothesis, we first determined the expression level and cellular location of β-catenin in SMMC-7721 cells transfected with miR-146a or miR-Ctrl. Western blotting analysis showed that the β-catenin level did not differ significantly between SMMC-7721 cells transfected with miR-146a and those transfected with the miR-Ctrl (Figure 4A). However, fluorescence microscopy analyses showed that β-catenin was found in both the nucleus (green) and cytoplasm of miR-Ctrl cells, however, overexpression of miR-146a resulted in the predominant localization of β-catenin in the plasma membrane (Figure 4C). Consistent with our fluorescence microscopy analyses, cellular fractionation showed that miR-146a overexpression inhibited the nuclear accumulation of β-catenin (Additional file 5: Figure S4A), indicating that miR-146a promotes cytoplasmic β-catenin accumulation through the APC pathway. In contrast, the opposite patterns of expression were detected in Huh-7 and HepG2 cells transfected with antagomiR-146a (Figure 4D, and Additional file 5: Figure S4B). In addition, we also found that the overexpression of miR-146a in SMMC-7721 cells resulted in reduced NF–κB expression levels (Figure 4A); immunofluorescence staining revealed that NF–κB protein was localized in cytoplasm (Figure 5G), however, the cells transfected with miR-Ctrl showed NF–κB mainly localized in prenuclear and nucleus region (Figure 5G). In contrast, miR-146a downregulation relocated NF–κB from the cytoplasm to the prenuclear and nucleus in Huh-7 cells transfected with antagomiR-146a (Figure 5H). The ectopic expression of either β-catenin or NF–κB in miR-146a-expressing SMMC-7721 cells reversed the ability of miR-146a to inhibit VEGFA expression and repress cells invasion (Figure 4E, and Additional file 6: Figure S7B). These results indicate that miR-146a represses VEGFA though the β-catenin and NF–κB signaling pathways.

Having established the downregulation of VEGFA by miR-146a *in vitro*, we next tested whether miR-146a would suppress VEGFA expression and angiogenesis in an animal model. We performed immunohistochemistry to detect VEGFA expression in tumors from miR-Ctrl and miR-146a-overexpressing mice and found that the control SMMC-7721 tumors showed higher levels of VEGFA staining. In contrast, miR-146a-expressing SMMC-7721 tumors showed only low levels of VEGFA staining (Figure 4F). Furthermore, CD34, a specific marker of endothelial cells, was used to detected angiogenesis. As shown in Figure 4F, reduced tumor microvessel density was observed in the miR-146a-expressing SMMC-7721 tumors compared to the control tumors (P = 0.0258). In addition, plasma VEGF levels were found to be decreased by 2.18 fold in mice with miR-146a compared with those in mice with miR-Ctrl (Additional file 7: Figure S5). Taken together, miR-146a affects tumor angiogenesis.

### HAb18G is a direct target of miR-146a in HCC cells

To understand the molecular mechanisms by which miR-146a induces tumor invasion and metastasis, we employed a tri-pronged approach to identify the potential downstream mRNA target(s) of miR-146a. First, we analyzed the upregulated gene expression profile in the Gene Expression Omnibus (GEO) database (GSE22058), which contains both miRNA and mRNA microarray data from 96 paired Hepatitis B Virus (HBV)-related HCC and adjacent non-cancerous tissues. Second, we focused on the mRNAs with increased expression in HCC tissues with metastasis, which we obtained from another dataset (GSE364). Third, we performed database searches in miRNA target prediction programs (Targetscan). *Ehmt2*, *mpzl1*, *rhoA*, *kif2c*, and *HAb18G* were chosen for further analysis because they are metastasis-related genes that are frequently overexpressed in HCC tissues and also contain a 3′ UTR element that is partly complementary to miR-146a.

To determine whether these 5 candidate mRNA transcripts are regulated by miR-146a through direct binding to its 3′ UTR, the 3′-UTR target sites of the candidate mRNAs (either wild-type and mutant) were inserted downstream of the luciferase promoter in the reporter vector. For luciferase assays, the miR-146a mimic or scrambled control was co-transfected with different luciferase 3′ UTR constructs and pRL-tk into SMMC-7721 cells (Figure 5A). As shown in Figure 5B and Additional file 8: Figure S6A, luciferase levels were reduced by more than 50% in the presence of miR-146a with the wild-type HAb18G 3′-UTR. This effect was not observed when the miR-146a binding sites were mutated. Thus, HAb18G is a potential target of miR-146a. Downregulated luciferase activity was also observed with the wild-type *KIF2C* 3′-UTR (Additional file 8: Figure S6A), suggesting that HAb18G is not the only direct target of miR-146a.

The effect of miR-146a on the endogenous expression of HAb18G was subsequently evaluated, and we observed a clear reduction in endogenous HAb18G protein in SMMC-7721 cells that overexpressed miR-146a. On the contrary, reduced expression of miR-146a in HepG2 and Huh-7 cells led to the increased expression of HAb18G protein (Figure 5C). Collectively, these results demonstrate that miR-146a downregulates HAb18G expression at the post-transcriptional level by directly targeting the 3′-UTR of the gene; therefore, HAb18G is a *bona fide* target of miR-146a. To determine whether the miR-146a-mediated suppression of HCC invasion was mediated by HAb18G, we established SMMC-7721-miR-146a cells overexpressing HAb18G from expression vector without 3′-UTR (Figure 5D). Deletion of 3′-UTR of HAb18G had induced the invasiveness of miR-146a overexpressing cells (Figure 5E) and increased expression of VEGF and NF–Kb p65 (Additional file 6: Figure S7). In contrast, knockdown of HAb18G by siRNA led to decreased VEGF protein level, NF–κB p65 level (Figure 5G), and cells invasiveness (Figure 5E). However, no apparent change in APC expression (Figure 5F), nor decreased nuclear accumulation of β-catenin (Additional file 9: Figure S8) were related with HAb18G protein level.

These data indicate that HAb18G downregulation is necessary, but not sufficient, to mediate the miR-146a-induced reduction in human HCC cell invasive potential. Furthermore, HAb18G is a potential target of miR-146a, but other functional miR-146a targets remain to be identified.

### MiR-146a expression was associated with decreased HAb18G, VEGF and NF-κB, and longer overall non-metastatic status in human HCC

**Figure 6:**
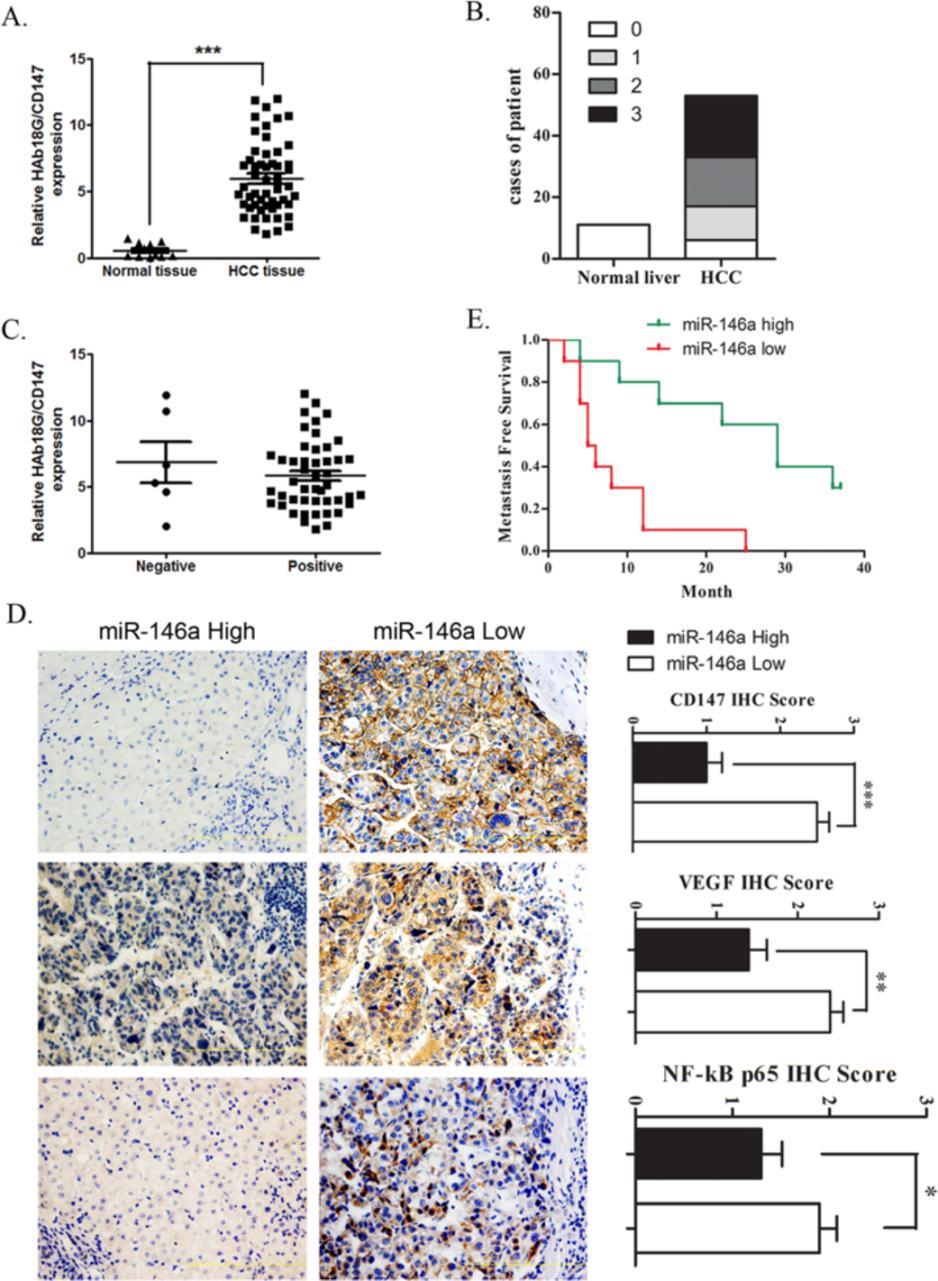
**Correlation between miR-146a and HAb18G/CD147, VEGF, NF-**κ**B p65 expression, and prognosis in HCC tissues. A**. Relative expression of HAb18G/CD147 in HCC tissue and in normal liver tissue by real-time RT-PCR. **B**. Staining score of HAb18G/CD147 in HCC tissue and in normal liver tissue by immunohistochemical staining. **C**. Relative expression of HAb18G/CD147 in immunohistochemical staining positive and negative HCC tissue by real-time RT-PCR. **D**. Representative images of immunohistochemical staining for HAb18G, VEGF and NF-κB p65 in low and high miR-146a expression cases (left). Scale bar represents 100 mm. Bar charts show the association between miR-146a expression and HAb18G, VEGF and NF-κB p65. The low and high miR-146a expression was stratified by using average of miR-146a expression as threshold. The y-axes represent HAb18G, VEGF and NF-κB p65 expression, as measured by immunohistochemical staining. Error bars represent ± SD. *p < 0.05, ** < 0.01. **E**. Kaplan-Meier metastasis-free survival curves in miR-146a low and high expression HCC cases. The low and high miR-146a expression was stratified by using average of miR-146a expression as threshold.

To extend our analysis to clinical cancers, we measured the expression of miR-146a in a cohort of 53 HCC samples (26 cases without metastasis, and 27 cases with metastasis) and 11 normal liver tissues. MiR-146a and HAb18G mRNA were measured using real-time PCR analysis in formaldehyde-fixed, paraffin-embedded tissues from the patients, and immunohistochemical staining for VEGF, NF-κB p65 and HAb18G were performed from the same patients. In comparison to normal liver tissues, the tumor samples showed an amplification of HAb18G mRNA and a significant downregulation of miR-146a expression (*P* < 0.001, Figure 1D, Figure 6A). Forty-seven tumors showed positive HAb18G staining and were classified as stage 1 (n = 11), stage 2 (n = 16), or stage 3 (n = 20) tumors. In addition, 6 tumors and 11 normal liver tissues were classified as stage 0 (Figure 6B). We also observed no significant difference in HAb18G mRNA expression between HAb18G-positive and HAb18G-negative HCC tissues (Figure 6C), suggesting that there may be post-transcriptional regulation of HAb18G expression at the protein level. Furthermore, miR-146a expression was inversely correlated with HAb18G, VEGF and NF-κB p65 protein expression (p = 0.0001, 0.0019, and 0.0453 respectively) (Figure 6D). We also found that NF-κB p65 mainly localized to perinuclear and nuclear region in low miR-146a expressed HCC tissues (Figure 6D). Remarkably, low miR-146a expression was significantly correlated with shorter non metastasis survival compared to those with high miR-146a expression (*P* < 0.001, Figure 6E).

## Discussion

Human miR-146a is embedded on chromosome 5q34, which is a region that is often deleted in HCC [13], and has been reported to be aberrantly expressed in several cancers. Other reports have shown that miR-146a functions as a tumor suppressive miRNA in several kinds of tumors, including pancreatic cancer, and breast cancers cancer [16, 30].

Our observation suggests that miR-146a was significantly downregulated in HCC cell lines as compared to normal liver tissue, which was consistent with our finding that miR-146a was expressed at lower levels in HCC tissues compared to adjacent non-cancerous tissues (Figure 1A). It may play a role in HCC tumoregenisis as a potential tumor suppressor. MiR-146a is also downregulated in metastatic HCC in comparison with non-metastatic HCC from patients (Figure 1F). These results suggest that miR-146a expression is often inhibited at earlier stages of cancer progression and that its expression level contributes to metastatic dissemination. Our observation also suggests that miR-146a is partially regulated by methylation. MiR-146a expression was restored in HCC cell lines upon administration of DNA methylation inhibitor 5-aza-2′-deoxycytidine (Figure 2A). Furthermore, we analyzed the methylation level of CpG sites in miR-146a promoter by bisulfite sequencing, our results suggest that the hypermethylation of the miR-146a promoter may be associated with down-expression of miR-146a in HCC tissues. Unfortunately, we could not reveal the apparent relationship involved in the methylation level and metastatic in HCC tissues in this study. However, it was notable that miR-146a promoter methylation level at one of seven the CpG site may related to metastatic (Figure 2D). Further studies using larger sample size are needed to reveal the relationship between miR-146a methylation and miR-146a expression, and HCC metastasis. MiR-146a was shown to directly inhibit expression of UHRF1 [31], an epigenetic regulator and coordinate tumor suppressor gene silencing via DNA methylation [32] in several cancers. Our results conjecture that repression of miR-146a may promote self-methylation by upregulation of UHRF1 in HCC. To identify this hypothesis, we compared the expression of UHRF1 mRNA by qRT-PCR and the methylation level by bisulfite sequencing in SMMC-7721 cells transfected with miR-Ctrl or miR-146a. The miR-146a treatment cells exhibited low expression of UHRF1 and lower methylation levels at seven CpG sites within the miR-146a promoter in comparison to the controls (Additional file 8: Figure S6C and D). These results hinted that there is a negative feedback loop in miR-146a expression. The further mechanism and function of this feedback regulation in HCC need to be further studied.

We testified the putative tumor suppressor function of miR-146a in human HCC by in vitro and in vivo assays. Ectopic expression of miR-146a could inhibit the invasion and metastasis of HCC cells (Figure 3B and C), and this effect was not due to suppressed cell cycle or increased apoptosis (Additional file 3: Figure S2). However, downregulated miR-146a was able to promote cell migration and invasion ability in 2 HCC cell lines (Huh-7 and HepG2) (Figure 3E and F). In nude mice, HCC cancer cells overexpressing miR-146a displayed significantly fewer metastatic loci than control cancer cells (Figure 3G,H and Additional file 2: Figure S1G). Collectively, our data indicate that miR-146a functions as a tumor suppressor in HCC. Our results imply that miR-146a may function as a negative regulator or tumor suppressor for cell invasion and metastasis in HCC.

The molecular mechanisms involved in miR-146a-mediated repression of metastasis are not well understood. In our study, we found that miR-146a inhibits HCC invasion and metastasis partly through the upregulation of APC and the downregulation of VEGF (Additional file 4: Figure S3 and Figure 4A). Adenomatous polyposis coli (APC), a tumor suppressor, interacts with β-catenin to inhibit Wnt signaling, is involved in multiple cellular functions and processes, including cell migration [33]. Intact APC exerts its anti-tumor effect through the accelerated degradation of β-catenin or by regulating β-catenin nuclear export and the inhibition of signal transduction by the lymphoid enhancer factor–T cell factor (LEF-TCF) family of transcription factors [34]. Here, we found that ectopic miR-146a expression increased APC protein levels and caused β-catenin to relocalize from the nucleus to the cytoplasm (Figure 4C,D, Additional file 5: Figure S4A and B). These findings indicate that miR-146a may inhibit the Wnt/β-catenin pathway by repressing nuclear β-catenin accumulation, which consequently leads to the partial downregulation of VEGF. However, the molecular mechanism involved in the upregulation of APC expression by miR-146a requires further investigation.

To date, several targets of miR-146a have been identified, such as IRAK-1 [17, 18]. EGFR [16], UHRF1 [31]. On the basis of our bioinformatics analysis and sequential experiments, we demonstrated that HAb18G is an additional miR-146a target in HCC cells. There are several lines of evidence to support this conclusion. First, based on our analysis using publicly available algorithms (TargetScan and miRanda), we found that HAb18G mRNA is a theoretical target gene of miR-146a, with potential binding sites conserved in multiple species (Additional file 8: Figure S6B). Importantly, our luciferase reporter assay results confirmed that miR-146a overexpression significantly downregulated luciferase activity by directly targeting the 3′-UTR of HAb18G mRNA. However, this effect was eliminated when the nucleic acids in the HAb18G 3′-UTR targeted by miR-146a were mutated. Second, HAb18G protein expression was significantly decreased in miR-146a-overexpressing SMMC-7721 cells as compared to the NC. Moreover, impaired miR-146a expression resulted in increased HAb18G protein levels in HepG2 and Huh-7 cells. Third, to further elucidate mechanisms underlying the tumor suppressive effect of miR-146a, we overexpressed HAb18G, but without its endogenous 3′-UTR in SMMC-7721-miR-146a cells and found that the invasion was increased, which suggest that the regulation of miR-146a on migration and invasion in HCC cells is related to HAb18G inhibition. We established the miR-146a-*HAb18G* axis by rescue experiment and the reverse correlation between miR-146a and HAb18G expression in HCC samples.

We observed decreased VEGF levels after HAb18G siRNA transfection and also found that elevated levels of HAb18G mRNA lacking the 3′-UTR were able to upregulate VEGF expression. Therefore, miR-146a could downregulate VEGF levels through the inhibition of HAb18G, leading to the suppression of cancer cell invasion and metastasis. However, the molecular mechanism involved in the upregulation of APC expression by miR-146a requires further investigation.

It has been reported that miR-146a can regulate NF-κB p65 signaling by targeting IRAK-1 [17, 18] or EGFR [16]. Importantly, we also observed decreased NF-κB p65 levels after reexpressing miR-146a or after HAb18G siRNA transfection (Figures 4A and 5F). Altogether, our data indicate that miR-146a can indirectly downregulate NF-κB p65 signaling by suppressing multiple molecules, resulting in the inhibition of cancer cell invasion and metastasis. On the other hand, Ectopic expression of NF–κB p65 or β-catenin in SMMC-7721-miR-146a cells reversed the effect of miR-146a to inhibit VEGFA expression (Figure 4E) and promote cell invasion (Additional file 6: Figure S7B). We also noted that decreased NF-κB p65 expression by siRNA could attenuate the HAb18G-induced SMMC-7721-miR-146a cells and repressed cells invasion (Additional file 6: figure S7C, D). These results indicate that miR-146a repress VEGFA though β-catenin and NF–κB signal pathway.

Moreover, we demonstrated that miR-146a downregulation in HCC cells led to the upregulation of VEGF via 2 signaling pathways: 1) the repression of APC expression, leading to β-catenin accumulation in nucleus, and 2) a direct reduction in HAb18G expression, which consequently promoted the expression of NF-κB p65. These findings will contribute to our understanding of the molecular mechanism by which miR-146a influences tumorigenesis and may aid in the development of novel cancer therapy strategies.

## Electronic supplementary material

Additional file 1: Table S1: primers and duplex. (XLSX 12 KB)

Additional file 2: Figure S1: miR-146a inhibited cell invasion and metastasis *in vitro* and *in vivo*. **A**. Represent figures of wound healing assay of SMMC-7721 cells transfected with miR-146a or miRNA control (miR-Ctrl). B-C. Represent figures of wound healing assay of Huh-7 **(B.)** and HepG2 **(C.)** cells transfected with antagomiR-146a or negative control (NC). D. Represent figures of invasion assay of SMMC-7721 cells transfected with miR-146a or miRNA control (miR-Ctrl). E-F. Represent figures of invasion assay of Huh-7 **(E.)** and HepG2 **(F.)** cells transfected with antagomiR-146a or negative control (NC). G. Number of visible lung metastases (n = 10 in each group). Data are present as mean + SD. * p < 0.05. (DOCX 1009 KB)

Additional file 3: Figure S2: Effect of miR-146a overexpression on HCC cell function. **A**. Flow cytometry analysis of the cell cycle in miR-Ctrl or miR-146a-transfected SMMC-7721 cells. **B**. Cell death was monitored using fluorescein isothiocyanate (FITC)-labeled AnnexinV and PI staining with flow cytometry. The right lower quadrant of each plot contains early apoptotic cells, whereas the right upper quadrant contains late apoptotic cells. This experiment was repeated 3 independent times, and similar results were obtained each time. **C**. Cell proliferation assay (miR-NC-transfected or miR-146a-transfected SMMC-7721 cells). **C**. Western blotting analysis of PCNA protein expression in miR-NC-transfected or miR-146a-transfected SMMC-7721 cells. Data represent the mean ± SEM of 3 independent experiments. **D**. Western blot analysis of PCNA in SMMC-7721 cell transfected with miR-Ctrl or miR-146a. (DOCX 485 KB)

Additional file 4: Figure S3: RT^2^ profiler PCR array data from human HCC cells SMMC-7721 cells transfected with miR-146a or miRNA control, displayed as a scatter plot. Genes differentially regulated by more than 2-fold are indicated. (DOCX 106 KB)

Additional file 5: Figure S4: Overexpression of miR-146a promote β-catenin localized in cytoplasm. **A**. Western blotting analysis of β-catenin expression in the cytoplasm and nuclei of SMMC-7721 cells transfected with miR-146a or miRNA control (miR-Ctrl). Nuclear protein Histone H3 was used as a nuclear protein marker, and GAPDH was used as a cytoplasmic protein marker. **B**. Western blotting analysis of β-catenin expression in the cytoplasm and nuclei of Huh-7 and HepG2 cell lines transfected with antagomiR-146a. Nuclear protein Histone H3 was used as a nuclear protein marker, and GAPDH was used as a cytoplasmic protein marker. (DOCX 313 KB)

Additional file 6: Figure S7: Relative expression of VEGFA and NF-κB p65 and in vitro invasion assay. **A**. Relative expression of VEGF and NF-κB in SMMC-7721-146a cells transfected with HAb18G Re. Error bars represent ± SD. *, p <0.05. **B**. In vitro invasion assay. SMCC-7721 cells transfected with pcDNA-VEGF or NF-κB p65 were seeded into triplicate mitrigel coated invasion chambers at 24 h post-transfection and allowed to invade toward serum for 24 h. The invading cell numbers on each filter were counted and data were plotted. The datum represents the mean ± SD. The student’s t test was used to compare the difference between two groups. *p < 0.05. **C**. Relative expression of NF-κB p65 in SMMC-7721-146a-HAb18G cells transfected with siRNA. Error bars represent ± SD. *, p <0.05. **D**. In vitro invasion assay. SMCC-7721-146a-HAb18G cells transfected with NF-κB p65 siRNA were seeded into triplicate mitrigel coated invasion chambers at 24 h post-transfection and allowed to invade toward serum for 24 h. The invading cell numbers on each filter were counted and data were plotted. The datum represents the mean ± SD. The student’s t test was used to compare the difference between two groups. *p < 0.05. (DOCX 225 KB)

Additional file 7: Figure S5: VEGF level released into the plasma of untreated, vector and miR-146a treated mice were detected by ELISA. The datum represents the mean ± SD. (DOCX 3 MB)

Additional file 8: Figure S6: Luciferase assay and Bisulfite genomic sequencing analysis. **A**. Luciferase activities of various reporter plasmids in HEK-293 T cells co-transfected with miR-146a or miR-Ctrl. Each experiment was performed in triplicate and the luciferase activity was shown as mean ± SD. A student t-test was employed. * refers to p < 0.05. **B**. Multiple species sequence alignment of the HAb18G/CD147 3′UTR including the putative miR-146a target site sequence (upper). **C**. Relative expression of UHRF1 in SMMC-7721 cells transfected with miR- Ctrl or miR-146a. Each experiment was performed in triplicate and the relative expression was shown as mean ± SD. A student t-test was employed. * refers to p < 0.05. **D**. Methylation status in SMMC-7721 cells transfected with miR- Ctrl or miR-146a. The filled and open boxes are represented methylated and unmethylated CpG, respectively. Five single clones are represented for each sample. Open boxes, 0% methylation; light filled boxes, 0-50% methylation; black filled boxes, 50-100% methylation. **E**. Represent figures of methylation status in SMMC-7721 cells transfected with miR- Ctrl or miR-146a. Red boxes shows methylation sites in miR-146a promoter. (DOCX 487 KB)

Additional file 9: Figure S8: Immunofluorescence staining of β-catenin (green) in SMMC-7721 cells transfected with siHAb18G. No differences in localization were observed. Right: Overlay of β-catenin (green) and nuclear DAPI (blue) staining. Scale bars, 50 μm. (DOCX 603 KB)

## Abbreviations

3′-UTR: 3′ untranslated region
APC: Adenomatous polyposis coli
HCC: Hepatocellular carcinoma
miRNA: microRNA
NC: negative control
siRNA: small interfering RNA
qPCR: quantitative polymerase chain reaction
SEM: Standard error of the mean.
