# Supplementary material for: microRNA-146a inhibits cancer metastasis by downregulating VEGF through dual pathways in hepatocellular carcinoma
Source: Mol Cancer. 2015 Jan 21;14:5. doi: 10.1186/1476-4598-14-5 (PMC4326400; doi:10.1186/1476-4598-14-5)
Supplement: Supplementary file 2 — Additional file 2: Figure S1: miR-146a inhibited cell invasion and metastasis in vitro and in vivo. A. Represent figures of wound healing assay of SMMC-7721 cells transfected with miR-146a or miRNA control (miR-Ctrl). B-C. Represent figures of wound healing assay of Huh-7 (B.) and HepG2 (C.) cells transfected with antagomiR-146a or negative control (NC). D. Represent figures of invasion assay of SMMC-7721 cells transfected with miR-146a or miRNA control (miR-Ctrl). E-F. Represent figures of invasion assay of Huh-7 (E.) and HepG2 (F.) cells transfected with antagomiR-146a or negative control (NC). G. Number of visible lung metastases (n = 10 in each group). Data are present as mean + SD. * p < 0.05. (DOCX 1009 KB) [file 12943_2014_1467_MOESM2_ESM.docx]

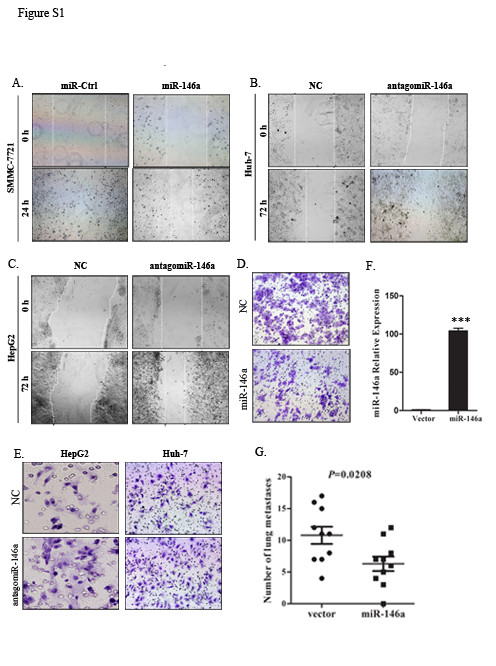


**Figure S1. miR-146a inhibited cell invasion and metastasis *in vitro* and *in vivo*.**

A. Represent figures of wound healing assay of SMMC-7721 cells transfected with miR-146a or miRNA control (miR-Ctrl).

B-C. Represent figures of wound healing assay of Huh-7(B.) and HepG2 (C.) cells transfected with antagomiR-146a or negative control (NC).

D. Represent figures of invasion assay of SMMC-7721 cells transfected with miR-146a or miRNA control (miR-Ctrl).

E-F. Represent figures of invasion assay of Huh-7(E.) and HepG2 (F.) cells transfected with antagomiR-146a or negative control (NC).

G. Number of visible lung metastases (n=10 in each group). Data are present as mean + SD. * p<0.05.
