# Supplementary material for: microRNA-146a inhibits cancer metastasis by downregulating VEGF through dual pathways in hepatocellular carcinoma
Source: Mol Cancer. 2015 Jan 21;14:5. doi: 10.1186/1476-4598-14-5 (PMC4326400; doi:10.1186/1476-4598-14-5)
Supplement: Supplementary file 3 — Additional file 3: Figure S2: Effect of miR-146a overexpression on HCC cell function. A. Flow cytometry analysis of the cell cycle in miR-Ctrl or miR-146a-transfected SMMC-7721 cells. B. Cell death was monitored using fluorescein isothiocyanate (FITC)-labeled AnnexinV and PI staining with flow cytometry. The right lower quadrant of each plot contains early apoptotic cells, whereas the right upper quadrant contains late apoptotic cells. This experiment was repeated 3 independent times, and similar results were obtained each time. C. Cell proliferation assay (miR-NC-transfected or miR-146a-transfected SMMC-7721 cells). C. Western blotting analysis of PCNA protein expression in miR-NC-transfected or miR-146a-transfected SMMC-7721 cells. Data represent the mean ± SEM of 3 independent experiments. D. Western blot analysis of PCNA in SMMC-7721 cell transfected with miR-Ctrl or miR-146a. (DOCX 485 KB) [file 12943_2014_1467_MOESM3_ESM.docx]

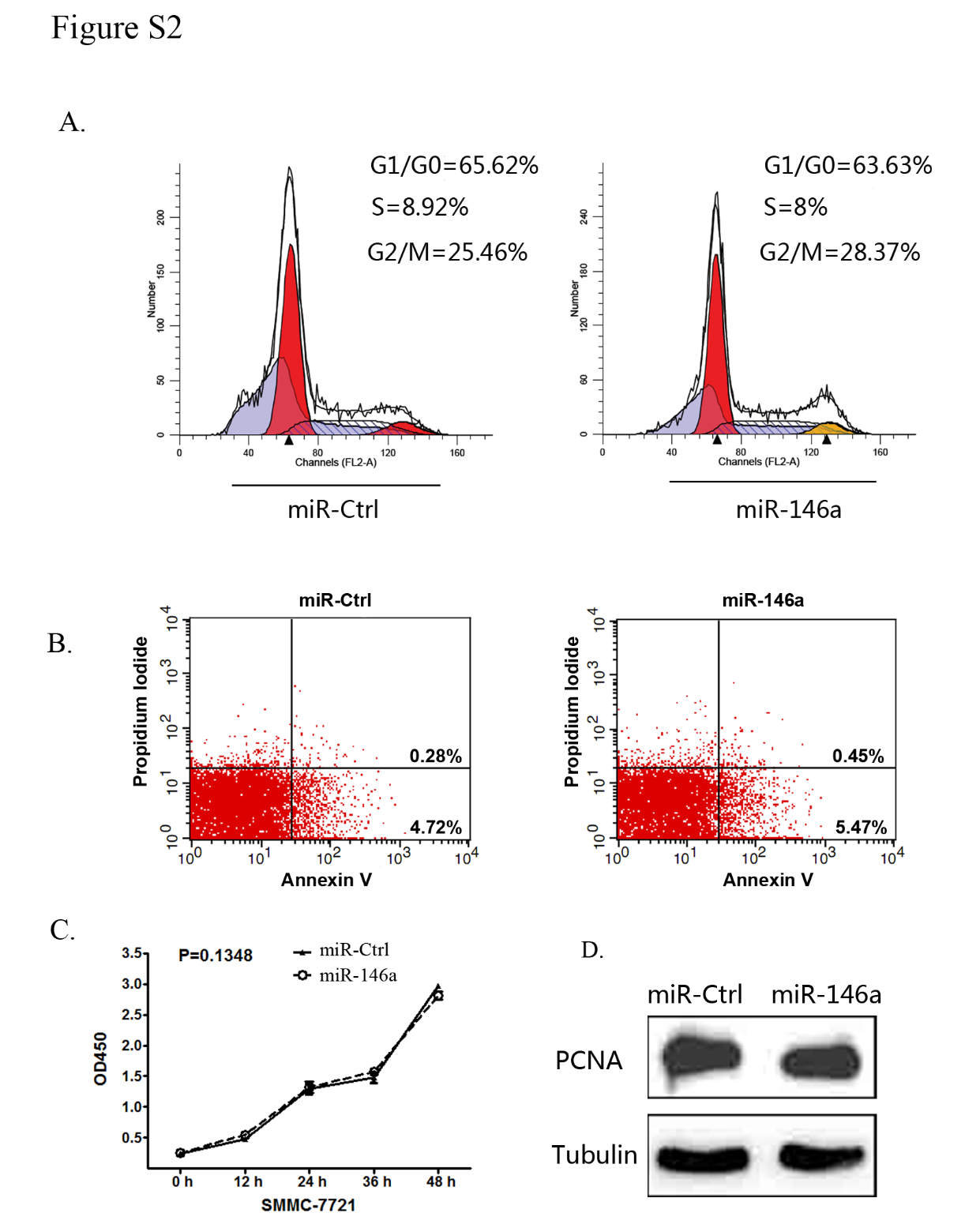


**Figure S2. Effect of miR-146a overexpression on HCC cell function.**

A. Flow cytometry analysis of the cell cycle in miR-Ctrl or miR-146a-transfected SMMC-7721 cells.

B. Cell death was monitored using ﬂuorescein isothiocyanate (FITC)-labeled AnnexinV and PI staining with flow cytometry. The right lower quadrant of each plot contains early apoptotic cells, whereas the right upper quadrant contains late apoptotic cells. This experiment was repeated 3 independent times, and similar results were obtained each time.

C. Cell proliferation assay (miR-NC-transfected or miR-146a-transfected SMMC-7721 cells). C. Western blotting analysis of PCNA protein expression in miR-NC-transfected or miR-146a-transfected SMMC-7721 cells. Data represent the mean±SEM of 3 independent experiments.

D. Western blot analysis of PCNA in SMMC-7721 cell transfected with miR-Ctrl or miR-146a.
