# Supplementary material for: microRNA-146a inhibits cancer metastasis by downregulating VEGF through dual pathways in hepatocellular carcinoma
Source: Mol Cancer. 2015 Jan 21;14:5. doi: 10.1186/1476-4598-14-5 (PMC4326400; doi:10.1186/1476-4598-14-5)
Supplement: Supplementary file 4 — Additional file 4: Figure S3: RT2 profiler PCR array data from human HCC cells SMMC-7721 cells transfected with miR-146a or miRNA control, displayed as a scatter plot. Genes differentially regulated by more than 2-fold are indicated. (DOCX 106 KB) [file 12943_2014_1467_MOESM4_ESM.docx]

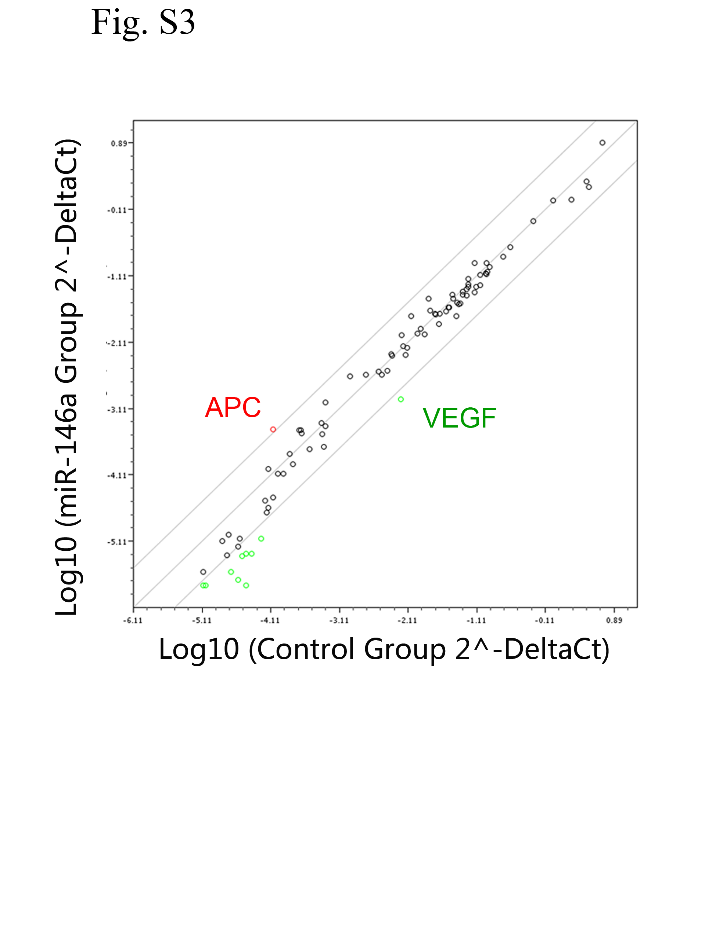


**Figure S3.** RT^2^ profiler PCR array data from human HCC cells SMMC-7721 cells transfected with miR-146a or miRNA control, displayed as a scatter plot. Genes differentially regulated by more than 2-fold are indicated.
