# Supplementary material for: microRNA-146a inhibits cancer metastasis by downregulating VEGF through dual pathways in hepatocellular carcinoma
Source: Mol Cancer. 2015 Jan 21;14:5. doi: 10.1186/1476-4598-14-5 (PMC4326400; doi:10.1186/1476-4598-14-5)
Supplement: Supplementary file 5 — Additional file 5: Figure S4: Overexpression of miR-146a promote β-catenin localized in cytoplasm. A. Western blotting analysis of β-catenin expression in the cytoplasm and nuclei of SMMC-7721 cells transfected with miR-146a or miRNA control (miR-Ctrl). Nuclear protein Histone H3 was used as a nuclear protein marker, and GAPDH was used as a cytoplasmic protein marker. B. Western blotting analysis of β-catenin expression in the cytoplasm and nuclei of Huh-7 and HepG2 cell lines transfected with antagomiR-146a. Nuclear protein Histone H3 was used as a nuclear protein marker, and GAPDH was used as a cytoplasmic protein marker. (DOCX 313 KB) [file 12943_2014_1467_MOESM5_ESM.docx]

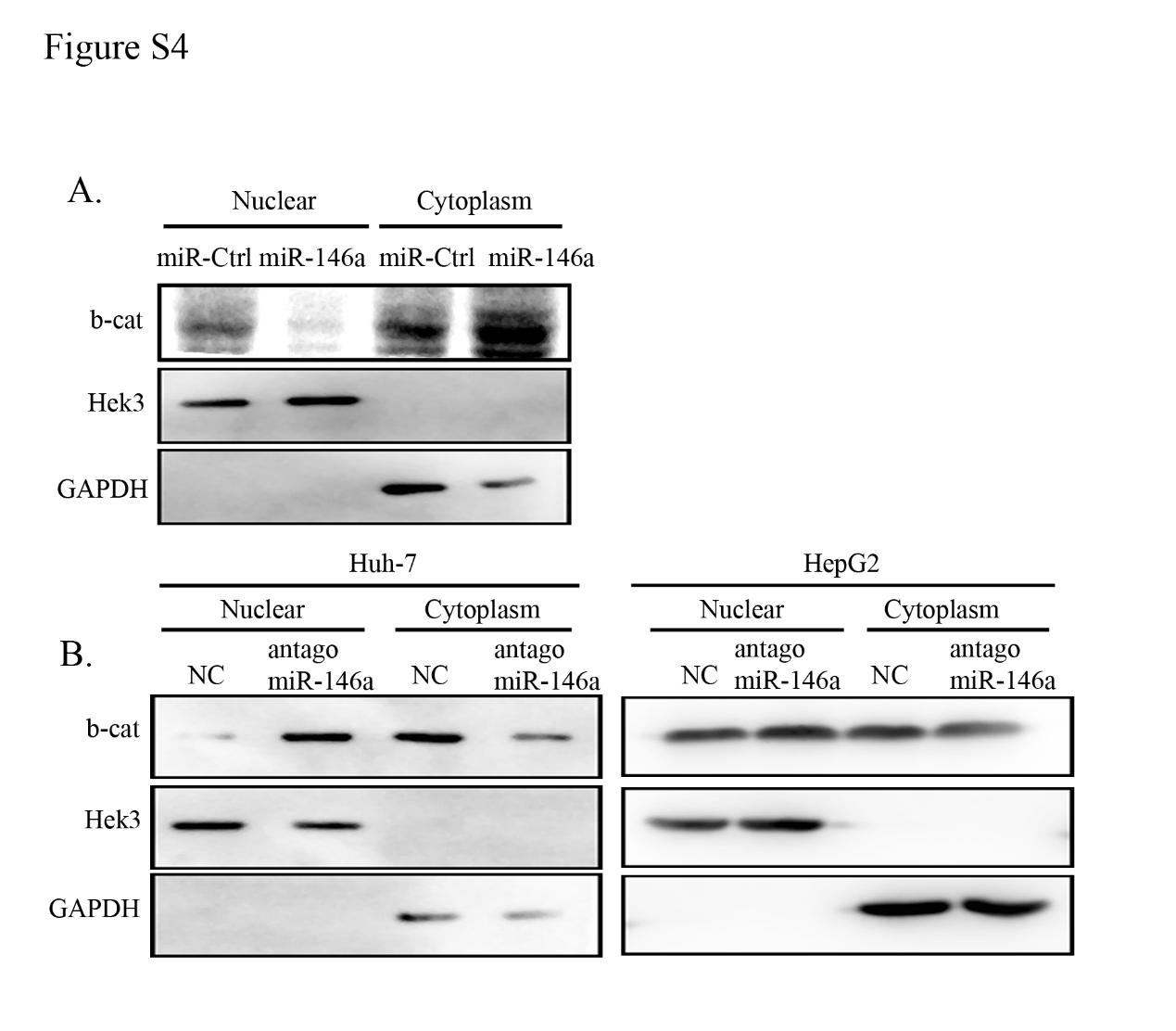


**Figure S4.** **Overexpression of miR-146a promote β-catenin localized in cytoplasm.**

A. Western blotting analysis of β-catenin expression in the cytoplasm and nuclei of SMMC-7721 cells transfected with miR-146a or miRNA control (miR-Ctrl). Nuclear protein Histone H3 was used as a nuclear protein marker, and GAPDH was used as a cytoplasmic protein marker.

B. Western blotting analysis of β-catenin expression in the cytoplasm and nuclei of Huh-7 and HepG2 cell lines transfected with antagomiR-146a. Nuclear protein Histone H3 was used as a nuclear protein marker, and GAPDH was used as a cytoplasmic protein marker.
