# Supplementary material for: microRNA-146a inhibits cancer metastasis by downregulating VEGF through dual pathways in hepatocellular carcinoma
Source: Mol Cancer. 2015 Jan 21;14:5. doi: 10.1186/1476-4598-14-5 (PMC4326400; doi:10.1186/1476-4598-14-5)
Supplement: Supplementary file 6 — Additional file 6: Figure S7: Relative expression of VEGFA and NF-κB p65 and in vitro invasion assay. A. Relative expression of VEGF and NF-κB in SMMC-7721-146a cells transfected with HAb18G Re. Error bars represent ± SD. *, p <0.05. B. In vitro invasion assay. SMCC-7721 cells transfected with pcDNA-VEGF or NF-κB p65 were seeded into triplicate mitrigel coated invasion chambers at 24 h post-transfection and allowed to invade toward serum for 24 h. The invading cell numbers on each filter were counted and data were plotted. The datum represents the mean ± SD. The student’s t test was used to compare the difference between two groups. *p < 0.05. C. Relative expression of NF-κB p65 in SMMC-7721-146a-HAb18G cells transfected with siRNA. Error bars represent ± SD. *, p <0.05. D. In vitro invasion assay. SMCC-7721-146a-HAb18G cells transfected with NF-κB p65 siRNA were seeded into triplicate mitrigel coated invasion chambers at 24 h post-transfection and allowed to invade toward serum for 24 h. The invading cell numbers on each filter were counted and data were plotted. The datum represents the mean ± SD. The student’s t test was used to compare the difference between two groups. *p < 0.05. (DOCX 225 KB) [file 12943_2014_1467_MOESM6_ESM.docx]

**
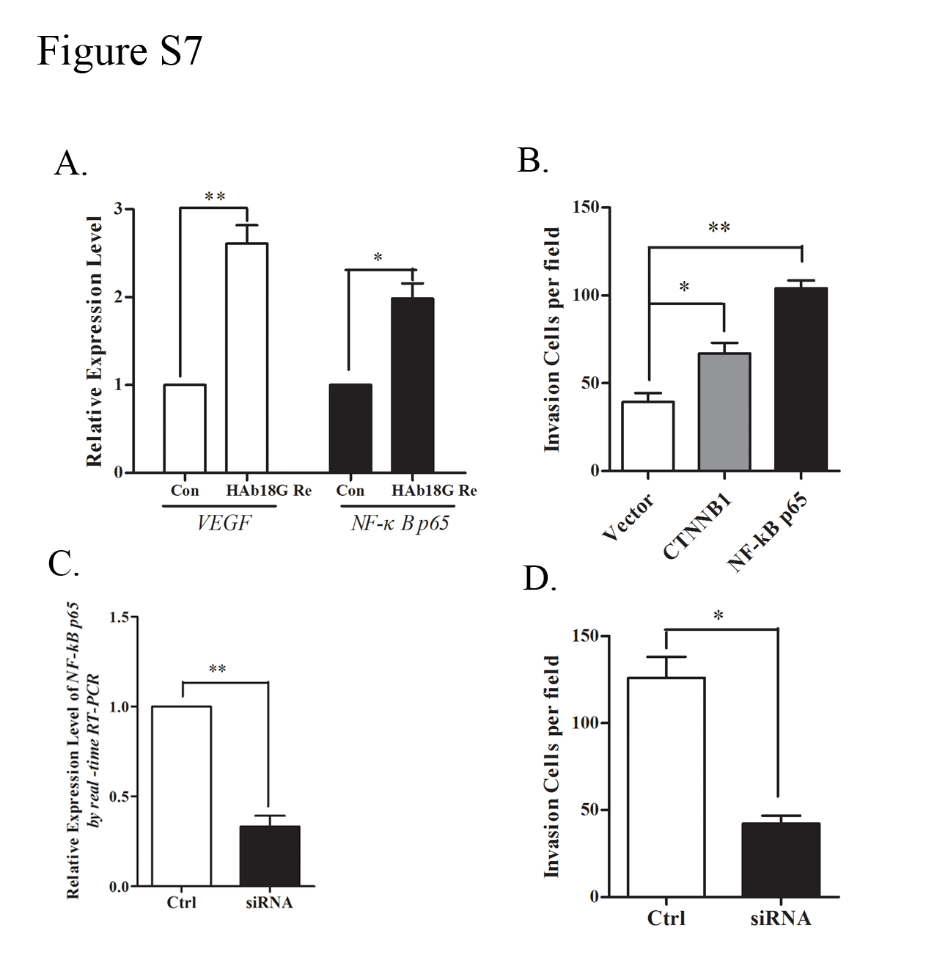
**

**Figure S7 Relative expression of VEGFA and NF-κB p65 and in vitro invasion assay.**

A. Relative expression of VEGF and NF-κB in SMMC-7721-146a cells transfected with HAb18G Re. Error bars represent ± SD. *, p <0.05.

B. In vitro invasion assay. SMCC-7721 cells transfected with pcDNA-VEGF or NF-κB p65 were seeded into triplicate mitrigel coated invasion chambers at 24 h post-transfection and allowed to invade toward serum for 24h. The invading cell numbers on each filter were counted and data were plotted. The datum represents the mean±SD. The student’s t test was used to compare the difference between two groups. *p<0.05.

C. Relative expression of NF-κB p65 in SMMC-7721-146a-HAb18G cells transfected with siRNA. Error bars represent ± SD. *, p <0.05.

D. In vitro invasion assay. SMCC-7721-146a-HAb18G cells transfected with NF-κB p65 siRNA were seeded into triplicate mitrigel coated invasion chambers at 24 h post-transfection and allowed to invade toward serum for 24h. The invading cell numbers on each filter were counted and data were plotted. The datum represents the mean±SD. The student’s t test was used to compare the difference between two groups. *p<0.05.
