# Supplementary material for: microRNA-146a inhibits cancer metastasis by downregulating VEGF through dual pathways in hepatocellular carcinoma
Source: Mol Cancer. 2015 Jan 21;14:5. doi: 10.1186/1476-4598-14-5 (PMC4326400; doi:10.1186/1476-4598-14-5)
Supplement: Supplementary file 7 — Additional file 7: Figure S5: VEGF level released into the plasma of untreated, vector and miR-146a treated mice were detected by ELISA. The datum represents the mean ± SD. (DOCX 3 MB) [file 12943_2014_1467_MOESM7_ESM.docx]

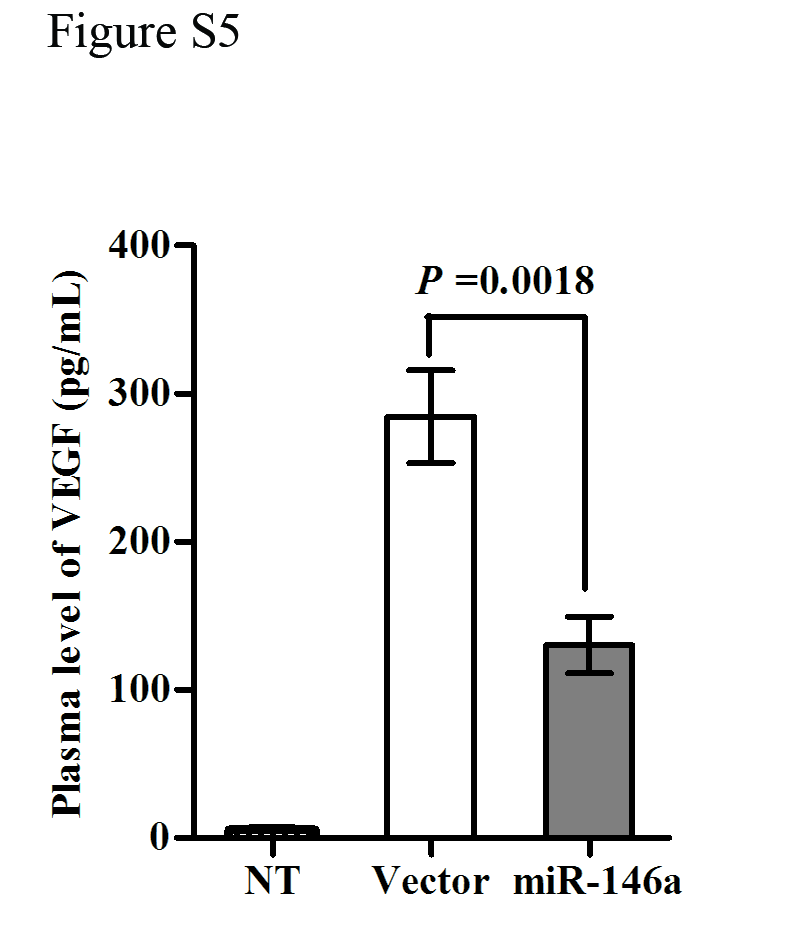


**Figure S5** VEGF level released into the plasma of untreated, vector and miR-146a treated mice were detected by ELISA. The datum represents the mean± SD.
