# Supplementary material for: microRNA-146a inhibits cancer metastasis by downregulating VEGF through dual pathways in hepatocellular carcinoma
Source: Mol Cancer. 2015 Jan 21;14:5. doi: 10.1186/1476-4598-14-5 (PMC4326400; doi:10.1186/1476-4598-14-5)
Supplement: Supplementary file 8 — Additional file 8: Figure S6: Luciferase assay and Bisulfite genomic sequencing analysis. A. Luciferase activities of various reporter plasmids in HEK-293 T cells co-transfected with miR-146a or miR-Ctrl. Each experiment was performed in triplicate and the luciferase activity was shown as mean ± SD. A student t-test was employed. * refers to p < 0.05. B. Multiple species sequence alignment of the HAb18G/CD147 3′UTR including the putative miR-146a target site sequence (upper). C. Relative expression of UHRF1 in SMMC-7721 cells transfected with miR- Ctrl or miR-146a. Each experiment was performed in triplicate and the relative expression was shown as mean ± SD. A student t-test was employed. * refers to p < 0.05. D. Methylation status in SMMC-7721 cells transfected with miR- Ctrl or miR-146a. The filled and open boxes are represented methylated and unmethylated CpG, respectively. Five single clones are represented for each sample. Open boxes, 0% methylation; light filled boxes, 0-50% methylation; black filled boxes, 50-100% methylation. E. Represent figures of methylation status in SMMC-7721 cells transfected with miR- Ctrl or miR-146a. Red boxes shows methylation sites in miR-146a promoter. (DOCX 487 KB) [file 12943_2014_1467_MOESM8_ESM.docx]

**
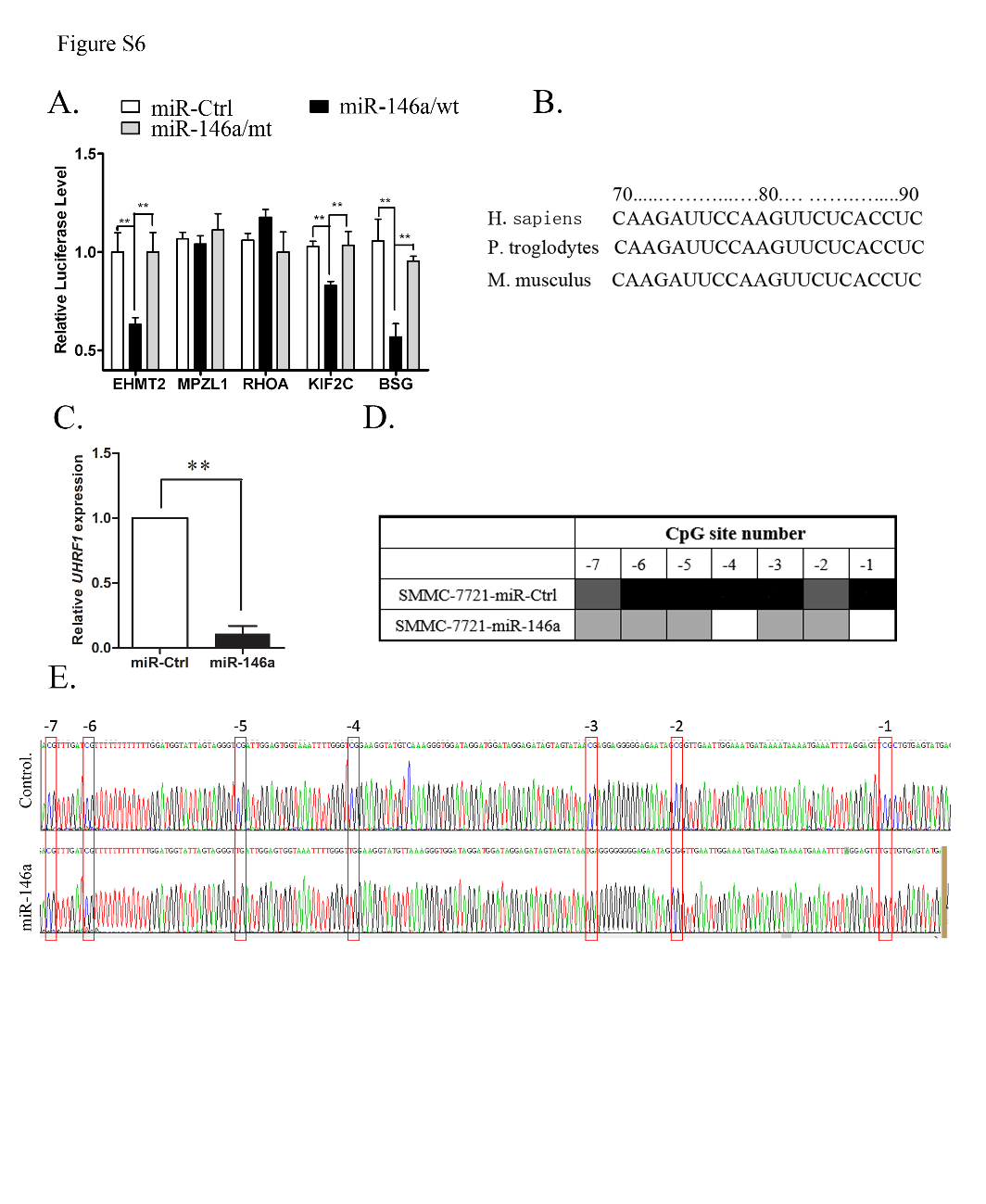
**

**Figure S6. Luciferase assay and Bisulfite genomic sequencing analysis.**

A. Luciferase activities of various reporter plasmids in HEK-293T cells co-transfected with miR-146a or miR-Ctrl. Each experiment was performed in triplicate and the luciferase activity was shown as mean ± SD. A student t-test was employed. * refers to p<0.05.

B. Multiple species sequence alignment of the HAb18G/CD147 3’UTR including the putative miR-146a target site sequence (upper).

C. Relative expression of UHRF1 in SMMC-7721 cells transfected with miR- Ctrl or miR-146a. Each experiment was performed in triplicate and the relative expression was shown as mean ± SD. A student t-test was employed. * refers to p<0.05.

D. Methylation status in SMMC-7721 cells transfected with miR- Ctrl or miR-146a. The filled and open boxes are represented methylated and unmethylated CpG, respectively. Five single clones are represented for each sample. Open boxes, 0% methylation; light filled boxes, 0-50% methylation; black filled boxes, 50-100% methylation.

E. Represent figures of methylation status in SMMC-7721 cells transfected with miR- Ctrl or miR-146a. Red boxes shows methylation sites in miR-146a promoter.
