# Supplementary material for: microRNA-146a inhibits cancer metastasis by downregulating VEGF through dual pathways in hepatocellular carcinoma
Source: Mol Cancer. 2015 Jan 21;14:5. doi: 10.1186/1476-4598-14-5 (PMC4326400; doi:10.1186/1476-4598-14-5)
Supplement: Supplementary file 9 — Additional file 9: Figure S8: Immunofluorescence staining of β-catenin (green) in SMMC-7721 cells transfected with siHAb18G. No differences in localization were observed. Right: Overlay of β-catenin (green) and nuclear DAPI (blue) staining. Scale bars, 50 μm. (DOCX 603 KB) [file 12943_2014_1467_MOESM9_ESM.docx]

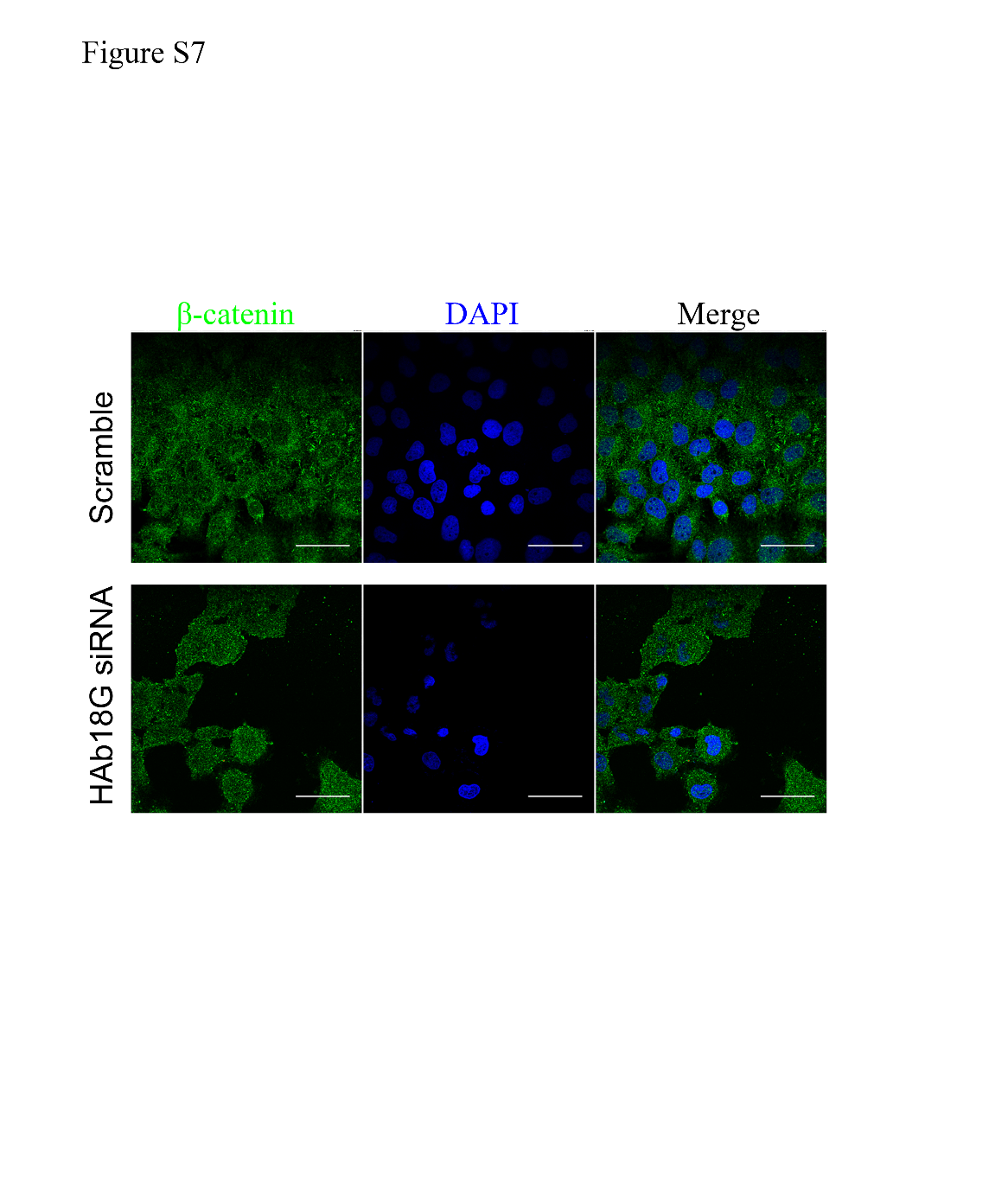


**Figure S8.** Immunofluorescence staining of β-catenin (green) in SMMC-7721 cells transfected with siHAb18G. No differences in localization were observed. Right: Overlay of β-catenin (green) and nuclear DAPI (blue) staining. Scale bars, 50 μm.
